# Transcriptomic and proteomic retinal pigment epithelium signatures of age-related macular degeneration

**DOI:** 10.1038/s41467-022-31707-4

**Published:** 2022-07-26

**Authors:** Anne Senabouth, Maciej Daniszewski, Grace E. Lidgerwood, Helena H. Liang, Damián Hernández, Mehdi Mirzaei, Stacey N. Keenan, Ran Zhang, Xikun Han, Drew Neavin, Louise Rooney, Maria Isabel G. Lopez Sanchez, Lerna Gulluyan, Joao A. Paulo, Linda Clarke, Lisa S. Kearns, Vikkitharan Gnanasambandapillai, Chia-Ling Chan, Uyen Nguyen, Angela M. Steinmann, Rachael A. McCloy, Nona Farbehi, Vivek K. Gupta, David A. Mackey, Guy Bylsma, Nitin Verma, Stuart MacGregor, Matthew J. Watt, Robyn H. Guymer, Joseph E. Powell, Alex W. Hewitt, Alice Pébay

**Affiliations:** 1 Garvan-Weizmann Centre for Cellular Genomics, Garvan Institute of Medical Research, Sydney, NSW 2010 Australia; 2 Department of Anatomy and Physiology, The University of Melbourne, Parkville, VIC 3010 Australia; 3 Centre for Eye Research Australia, Royal Victorian Eye and Ear Hospital, East Melbourne, VIC 3002 Australia; 4 Macquarie Medical School, Faculty of Medicine, Health and Human Sciences, Macquarie University, Sydney, NSW 2109 Australia; 5 QIMR Berghofer Medical Research Institute, Brisbane, QLD 4006 Australia; 6 Department of Cell Biology, Harvard Medical School, Boston, MA 02115 USA; 7 Lions Eye Institute, Centre for Vision Sciences, University of Western Australia, Perth, WA 6009 Australia; 8 School of Medicine, University of Tasmania, Hobart, TAS 7005 Australia; 9 Department of Surgery, Ophthalmology, Royal Victorian Eye and Ear Hospital, The University of Melbourne, East Melbourne, VIC 3002 Australia; 10 UNSW Cellular Genomics Futures Institute, University of New South Wales, Sydney, NSW 2052 Australia; 11 Menzies Institute for Medical Research, University of Tasmania, Hobart, TAS 7000 Australia; 12 Department of Surgery, Royal Melbourne Hospital, The University of Melbourne, Parkville, VIC 3010 Australia

**Keywords:** Induced pluripotent stem cells, Gene regulatory networks, Macular degeneration

## Abstract

There are currently no treatments for geographic atrophy, the advanced form of age-related macular degeneration. Hence, innovative studies are needed to model this condition and prevent or delay its progression. Induced pluripotent stem cells generated from patients with geographic atrophy and healthy individuals were differentiated to retinal pigment epithelium. Integrating transcriptional profiles of 127,659 retinal pigment epithelium cells generated from 43 individuals with geographic atrophy and 36 controls with genotype data, we identify 445 expression quantitative trait loci in cis that are asssociated with disease status and specific to retinal pigment epithelium subpopulations. Transcriptomics and proteomics approaches identify molecular pathways significantly upregulated in geographic atrophy, including in mitochondrial functions, metabolic pathways and extracellular cellular matrix reorganization. Five significant protein quantitative trait loci that regulate protein expression in the retinal pigment epithelium and in geographic atrophy are identified - two of which share variants with cis- expression quantitative trait loci, including proteins involved in mitochondrial biology and neurodegeneration. Investigation of mitochondrial metabolism confirms mitochondrial dysfunction as a core constitutive difference of the retinal pigment epithelium from patients with geographic atrophy. This study uncovers important differences in retinal pigment epithelium homeostasis associated with geographic atrophy.

## Abstract (web summary)

Age-related macular degeneration (AMD) is a leading cause of vision loss, and there is no approved treatment for AMD with geographic atrophy. Here, the authors used transcriptomic and proteomic analyses of patient induced pluripotent stem cell-derived retinal pigment epithelium to better understand disease mechanisms.

## Introduction

Age-related macular degeneration (AMD) is a progressive, degenerative disease caused by dysfunction and death of the retinal pigment epithelium (RPE), and photoreceptors, leading to irreversible vision loss. AMD is the leading cause of vision loss and legal blindness in higher-resourced countries^1^. There are two forms of the vision-threatening late stage of AMD; neovascular and geographic atrophy^2^, the latter affecting more than 5 million people globally^3^. Whilst management of neovascular AMD has improved significantly since the introduction of intravitreal antivascular endothelial growth factor (VEGF) injections^4–7^, there are currently no approved or effective treatments for geographic atrophy, despite multiple clinical trials to evaluate potential drug candidates and interventions^8–13^. This presents a significant unmet medical need and as such, greater effort in disease modeling and drug discovery should be aimed at preventing and delaying disease progression.

It is now well established that both environmental and genetic risk factors contribute to AMD^14,15^. To date, genome-wide association studies (GWAS) have identified 90 independent variants over 55 loci where a common risk allele is associated with an increased risk of AMD^16–22^, with those identified  by Fritsche et al.^18^ explaining over half of the genomic heritability. These loci influence distinct biological pathways, including the complement system, lipid transport, extracellular matrix remodeling, angiogenesis, and cell survival^23^.

Unlike rare and highly penetrant variants that largely contribute to disease by altering protein sequences, common variants predominantly act via changes in gene regulation^24^. Mapping expression quantitative trait loci (eQTL) is a powerful approach to elucidate functional mechanisms of common genetic variants, allowing the allelic effect of a variant on disease risk to be linked to changes in gene expression. Three recent studies applied eQTL mapping in the postmortem retina to investigate the regulation of gene expression and identified eQTL variants regulating gene expression with a subset of these eQTL associated with AMD in GWAS^25–27^. Molecular and genetic profiling of RPE in healthy and diseased tissue would likely improve our understanding of the mechanisms that confer disease risk or contribute to geographic atrophy progression. However, the invasive nature of retina harvest highly restricts tissue availability to postmortem donors. This limitation can be overcome by reprogramming somatic cells from affected patients into patient-specific induced pluripotent stem cells (iPSCs)^28,29^ and subsequently differentiating them into homogenous RPE cultures for downstream disease modeling. iPSC-derived RPE cells have been widely used to demonstrate disease phenotypes and elucidation of disease mechanisms such as those observed in macular dystrophies^30^ and in degenerative diseases including AMD^31–38^. Patient iPSC-derived cells, including RPE cells, can also be subjected to single-cell RNA-sequencing (scRNA-seq) to precisely characterize individual cells based on their unique transcriptional signature, identify subpopulations of cells within a cell culture and examine rare populations that would be missed using bulk RNA-seq, given its narrower resolution^39–42^. Using this approach, in conjunction with mass spectrometry, we characterized the transcriptomic and proteomic profiles of iPSC-derived RPE cells from a large cohort of people with or without geographic macular degeneration, the advanced form of AMD. This allowed us to compare samples at the phenotypic end of AMD with samples from unaffected controls. eQTL mapping implicated genes at loci definitively associated with disease, whilst also uncovering many new pathogenic loci and pathways. The functional analysis confirmed that identified pathways were dysregulated in geographic atrophy RPE cells.

## Results

### Generation of patient iPSCs, differentiation to RPE cells, and genomic profiling

**Fig. 1:**
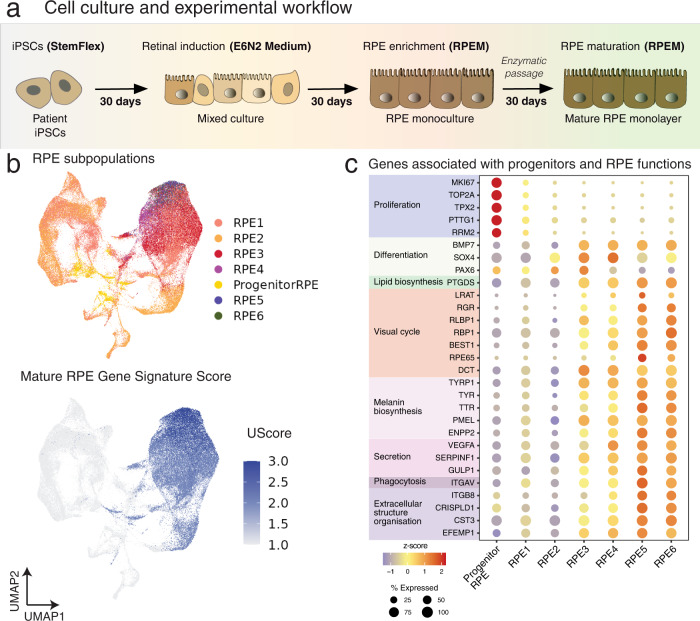
Generation of RPE from iPSCs, identification, and characterization of RPE subpopulations. **a** Schematic representation of the cell culture flow, with iPSCs differentiated into RPE cells in 90 days prior to harvest for scRNA-seq and mass spectrometry. **b** Uniform Manifold Approximation and Projection (UMAP) of cells labeled by subpopulation and RPE UScore. Cells were assigned to subpopulations identified in a previous study using a supervised classification method, and colored by subpopulation, and similarity to adult RPE was calculated using the UScore R package. **c** Dotplot representation of average expression following z-transformation of genes associated with RPE functions (extracellular structure organization, phagocytosis, secretion, melanin biosynthesis, visual cycle, lipid biosynthesis, differentiation, and proliferation) and progenitors (differentiation, proliferation) in the various subpopulations. Levels of gene expression per cell are shown with color gradients, and frequencies of cells expressing the respective gene (% expressed) are shown in size of dots.

We reprogrammed fibroblasts into iPSCs from 63 individuals with geographic atrophy (all of Northern European descent, 37 female and 26 male; mean ± SD age at recruitment: 83.8 ± 8.2 years) using episomal vectors as described in ref. ^43^, with lines from 47 individuals successfully reprogrammed (Supplementary Figs. 1 and 2). We matched these iPSCs with control iPSC lines from ethnically matched healthy individuals that were generated and characterized in a previous study^44^ (Supplementary Figs. 1 and 2, and Supplementary Data 1). Each line was genotyped for 787,443 single nucleotide polymorphisms (SNPs) and imputed with the Haplotype Reference Consortium panel^45^. After quality control, this yielded 4,309,001 autosomal SNPs with MAF > 10%. Disease-associated haplotypes in the *ARMS2-HTRA1* locus were present in 9 geographic atrophy patients and absent in controls (Supplementary Data 1). The *CFH Y402H* polymorphism (rs1061170) was present in 13 geographic atrophy patients and 1 control patient (Supplementary Data 1). The differentiation of all iPSC lines to RPE was performed in two large independent differentiation batches, and lines that did not differentiate sufficiently to RPE were discarded from analysis, which was based on clear cobblestone morphology of the cells and pigmentation throughout the culture (Fig. 1a and Supplementary Figs. 1–3). This method of differentiation has already been demonstrated as generating functionally mature RPE cells that resemble their native counterpart, in terms of expression of key RPE markers and functional assays^42,46^, and illustrated in Supplementary Fig. 3 with the expression of ZO-1, PMEL, BESTROPHIN, RPE65. Transepithelial electrical resistance readings confirmed the RPE cell barrier integrity and permeability, consistent with the presence of functional tight junctions in the RPE (Supplementary Fig. 3). Differentiated cell lines were divided into 12 pools that each consisted of up to eight cell lines from both control and AMD groups. scRNA-seq was performed on all pools, with the targeted capture of 20,000 cells per pool and a sequencing depth of 30,000 reads per cell (Supplementary Table 1). The resulting single-cell transcriptome profiles then underwent quality control and donor assignment. In all, 18,820 cells were designated doublets and removed from the data in addition to cells from individuals that were removed due to a low number of assigned cells (4), failed virtual karyotype (1) and failed genotype (4) (Supplementary Fig. 2 and Supplementary Table 2). A total of 127,659 cells from 79 individual lines remained following quality control. These include 43 geographic atrophy lines (73,161 cells, 15 males, 28 females, 83.4 ± 8.6 years) and 36 control lines (54,498 cells, 19 males, 17 females, mean ± SD age of samples 67.6 ± 9.5 years) (Supplementary Fig. 2 and Supplementary Data 1).

### Identification of seven RPE subpopulations using supervised classification

We previously used scRNA-seq to analyze the transcriptomic signature of a pure human embryonic stem cell-derived culture of RPE cells over 12 months in culture and identified 17 RPE subpopulations of varying levels of maturity^42^. This resource was used to build a prediction model for *scPred*, a supervised classification method^47^. While all 17 reference subpopulations were detected in this dataset, five subpopulations had fewer than 20 cells and were excluded from further analysis (Supplementary Table 3). Cells from donors with fewer than 20 cells in a subpopulation were also excluded. This left 127,659 cells (54,498 control, 73,161 geographic atrophy cells) distributed among the remaining 7 subpopulations, with cells being classified as “RPE progenitors” (Progenitor RPE) and RPE cells (RPE1-6) (Supplementary Table 1, Fig. 1, and Supplementary Data 1). A Chi-Squared Test of Independence observed statistically significant differences in the proportions of subpopulations between cases and controls (*X*^2^ (6, *N* = 127,659) = 3672.4, *P* < 2.2 × 10^−16^, Table 1). Post hoc pairwise comparisons revealed that the proportion of all subpopulations except RPE1 differed between cases and controls (Supplementary Table 4), and there was also variation in the proportions of subpopulations between individual cell lines (Supplementary Table 5 and Supplementary Fig. 4).

**Table 1:** Summary of cells retrieved in each RPE subpopulation.

Subpopulation	Number of cells (control)	Number of cells (GA)	Total number of cells	% of control cells	% of GA cells
Progenitor RPE	3159	2977	6136	5.8%	4.1%
RPE1	24,027	32,254	56,281	44.1%	44.1%
RPE2	16,780	14,620	31,400	30.8%	19.9%
RPE3	5851	14,501	20,352	10.7%	19.8%
RPE4	2113	4329	6442	3.9%	5.9%
RPE5	1396	2269	3665	2.6%	3.1%
RPE6	1172	2211	3383	2.1%	3.0%
Total	54,498	73,161	127,659	100.0%	100.0%

*RPE* retinal pigmented epithelium, *GA* geographic atrophy.

The transcriptomic variations observed within the RPE subpopulations were reflective of changes in maturity, rather than in cell identity, which is consistent with our previous work^42^. The gene expression pattern of the subpopulations indicates a continuum of differentiation, starting with progenitor cells (Progenitor RPE, expression of genes associated with cell proliferation^42^), cells that reached an RPE phenotype (RPE1 and RPE2, expression of canonical RPE genes, and genes involved in maturation) and RPE cells with expression of more advanced characteristics (RPE3 to RPE6, the highest expression of canonical RPE genes) (Fig. 1c and Supplementary Data 1), as observed in other studies^48^. The expression of the retinal development marker *PAX6*, known to contribute to RPE melanogenesis^49^, in the RPE subpopulations also suggests a continuing maturation and pigmentation of RPE cells^48^ (Fig. 1c). Various RPE markers were observed in all RPE subpopulations, albeit with different levels of expression, confirming the RPE identity of the cells as well as a continuum of maturation within the culture. These include genes associated with extracellular structure organization (*CST3*, *EFEMP1*, *ITGAV*, *CRISPLD1*, *ITGB8*), phagocytosis (*GULP1*), secretion (*SERPINF1*, *VEGFA*, *ENPP2*), melanin biosynthesis (*PMEL*, *TTR*, *TYR*, *TYRP1*, *DCT*), visual cycle (*RPE65*, *BEST1*, *RBP1*, *RLBP1*, *RGR, LRAT*), and lipid biosynthesis (*PTGDS*) (Fig. 1c and Supplementary Data 1). For instance, the canonical RPE markers *EFEMP1*, *ITGAV*, *CRISPLD1*, *ITGB8*, *GULP1*, *SERPINF1*, *VEGFA*, *ENPP2*, *PMEL*, *TYRP1*, *DCT,* and *RBP1* were already expressed by RPE1 and RPE2; *TYR*, *BEST1*, *RLBP1* in RPE1; and *CST3* by RPE2 (Fig. 1c and Supplementary Data 1), further confirming that these subpopulations have reached an RPE phenotype.

To determine how transcriptionally similar these cells were to adult RPE^50^, we defined the RPE gene signature using RPE-specific markers curated from a reference adult RPE dataset^50^, and measured their enrichment in each cell using gene signature analysis^51^ (Supplementary Data 1). We compared the distribution of scores in each subpopulation to a reference range we defined by measuring the signal in iPSCs from our previous work^41^, and cells of the peripheral retina and retinal organoids from ref. ^52^ (Supplementary Fig. 4b). The RPE gene signature was strongest in RPE5 (mean UScore: 0.44 ± 0.05) and weakest in the Progenitor RPE subpopulation (mean UScore: 0.19 ± 0.07). The bimodal distribution of scores in Progenitor RPE, RPE1, and RPE2 indicated the presence of cells with an emerging mature RPE gene signature (Supplementary Fig. 4b). Together with the characterization of key RPE protein expression and functions (Supplementary Fig. 3), these data confirm the cells in culture are a valid model for the study of their in vivo counterparts.

### Profiling of geographic atrophy-related expression patterns in RPE and RPE subpopulations

**Fig. 2:**
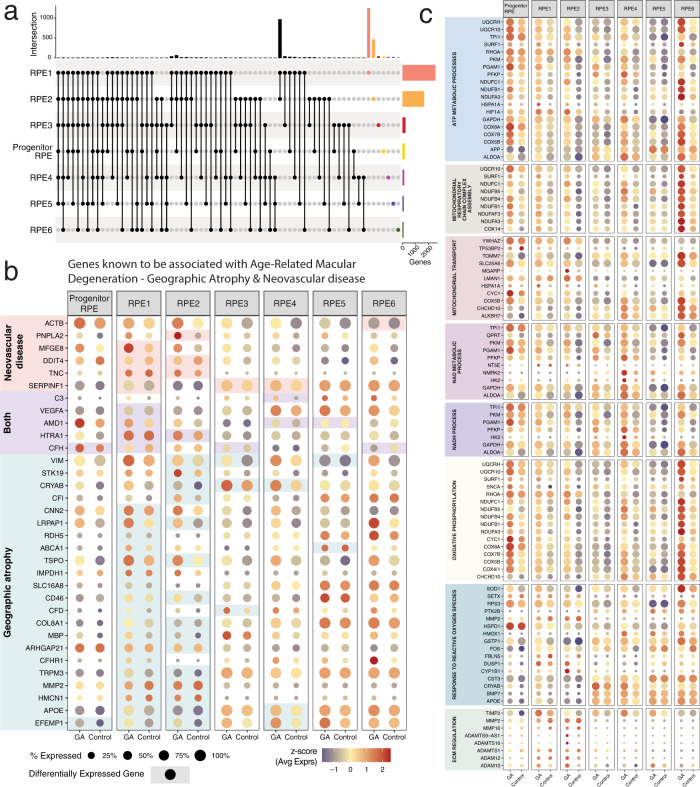
Genes associated with cell subpopulations and their expression in geographic atrophy and control cells. **a** Summary of cell subpopulation- specific gene regulation. **b**, **c** Dotplot representation of single-cell expression profile of genes associated with geographic atrophy or neovascular AMD (**b**) and of genes associated with biological processes of interest (**c**) in geographic atrophy (GA) and in control iPSC-derived subpopulations. Dotplots in **b** and **c** represent scaled average expression (z-scores) of genes, and percentage of cells in the cluster that express the gene in each subpopulation and condition. Blue end of the color scale represents below-average expression, and the red end of the scale represents above-average expression. Levels of gene expression per cell are shown with color gradients, and frequencies of cells expressing the respective gene (% expressed) are shown with size of dots. In b, significance is shown with colored background.

Next, we leveraged the power of analysis afforded by our large-scale experimental setup to identify gene expression profiles that are associated with the disease status in each RPE subpopulation on a cellular level. For this purpose, we used differential gene expression analysis and over-representation analysis. First, we compared the expression of cells from all geographic atrophy lines to cells from all control lines, and identified 3911 differentially expressed (DE) genes (Adjusted *P* value *<* 7.69 × 10^−6^) (Supplementary Data 2). The majority of DE genes were upregulated in geographic atrophy (78.4%) and consisted of protein-coding genes (3855), long noncoding RNA (37) and antisense RNA (19). We repeated this analysis within each subpopulation to review disease-associated expression in the context of cell state, and identified an additional 557 DE genes (Table 2 and Supplementary Data 2) that were predominantly specific to a single subpopulation (Fig. 2a). These genes were also mostly protein-coding (651), followed by long noncoding RNA (14) and antisense RNA (6). In all, 27 genes had known associations with geographic atrophy, and six had known associations with neovascular AMD; examples include *C3*^53^, *CFH*^54^, *CFHR1*^55^, *APOE*^56^, and *HTRA1*^57^ (Fig. 2b).

**Table 2:** Summary of differentially expressed genes in geographic atrophy (GA).

Subpopulation	Upregulated	Downregulated	Total	Replication rate—Dhirachaikulpanich et al.^58^—macula (%)	Replication rate—Dhirachaikulpanich et al.^58^—non-macula (%)	Replication rate—Kim et al.^60^ (%)	Known Association with GA
Progenitor RPE	54	131	185	0.1	0	0.6	2
RPE1	1987	578	2565	1.9	1.0	16.6	22
RPE2	1361	328	1689	1.5	0.6	11.2	15
RPE3	156	88	244	0.3	0.1	1.8	4
RPE4	126	15	141	0.3	0	1.5	5
RPE5	36	68	104	0.2	0.1	0.3	3
RPE6	41	43	84	0	0	0.5	1

We compared our results to 1139 DE genes (macula: 895, non-macula: 540) identified in ref. ^58^ in a meta-analysis of a microarray dataset in ref. ^59^ and a bulk RNA-seq dataset in ref. ^26^ that both feature samples of the RPE-choroid from the macula and non-macula, and 4499 DE genes from a study in ref*.* ^60^ which profiled the transcriptomes of peripheral RPE-choroid-sclera with bulk RNA-seq (Supplementary Table 6 and full results in Supplementary Data 2). Both studies were generated from postmortem samples of healthy individuals and those with AMD at different stages. We examined the intersection of our results with the results from these bulk studies and calculated the replication rate for each bulk study. The replication rate was low across all studies (ref*.* ^58^—macula: 2.5%, ref. ^58^—Non-macula: 1.8%, ref. ^60^ 23.4%), that is likely due to the majority of genes from the single-cell-level DE analysis being upregulated in geographic atrophy. Genes common to this study and the bulk studies displayed a bias towards upregulation in geographic atrophy (ref. ^58^—macula: 74.8%, ref. ^58^—Non-macula: 82.2%, ref. ^60^ 77.0%). We repeated this analysis to determine if this trend continued with geographic atrophy-associated genes discovered at a subpopulation level, and continued to observe low replication rates across all subpopulations (Table 2 and full results in Supplementary Table 6). We did not observe bias toward upregulation in three subpopulations—Progenitor RPE (27.4%), RPE5 (42%) and RPE6 (51.9%), and these subpopulations did not display bias towards upregulation when all DE genes were considered (Table 2). Subpopulation-level DE genes did not exhibit a bias towards results from region-specific bulk DE results; our results replicated more genes from the macula than the non-macula in the study in ref. ^58^, but replicated even more genes identified in the study in ref. ^60^ that profiled RPE-choroid-sclera from the periphery.

The bias toward upregulation of genes in geographic atrophy in our study contributed to the low replication rate of genes from bulk RNA studies, so we reviewed the composition and number of individuals recruited for each study to see if this was the cause. Our study had more individuals with geographic atrophy than healthy individuals (Geographic atrophy: 63, Healthy: 47), and subsequently there were more cells from geographic atrophy lines than healthy lines (Geographic atrophy: 73,161 cells, Healthy: 54,498 cells). The study in ref. ^60^ had the least number of participants with only eight healthy and eight individuals with AMD, and half of the individuals with AMD had late dry AMD which also included one donor with RPE atrophy. Orozco et al.^26^ did not differentiate between the different forms of AMD in the 23 donors with the condition, and the study in ref. ^59^ recruited two individuals with geographic atrophy and 17 with dry AMD. As all bulk studies had a mixed AMD cohort, it is unlikely these differences in replication are due to disease progression. This leaves sample size as a possible explanation for the higher replication rate of our results in the study in ref. ^60^; according to a study in ref. ^61^, studies with smaller sample sizes had greater variation in the number of transcripts quantified for each gene.

We identified genes belonging to pathways identified in ref. ^58^ that were also known to play roles in retinal biology and in AMD^62^, such as those related to extracellular cellular matrix (ECM) reorganization—matrix metalloproteinases (MMP) *MMP2* and *MMP16*, tissue inhibitors of metalloproteinases (TIMP)—*TIMP2* and *TIMP3*, a disintegrin and metalloproteinase domain (ADAM)—*ADAM10* and *ADAM12*, and various cADAM with thrombospondin motifs (ADAMTS) (Fig. 2c and Supplementary Data 2). Genes from the complement system are known to have a role in AMD^63^, so we reviewed if these genes^64^ were differentially expressed within our subpopulations. We identified four genes related to the activation of the complement pathway (*CFD*, *C1R*, *C1S,* and *C3*), and six genes from the regulation of the complementation pathway (*CFH*, *CLU*, *CFHR1*, *CD46*, *SERPING1*, *CFI*, and *CD55*).

We used over-representation analysis^65^ to identify which pathways related to diseases and biological functions were enriched in DE genes of each subpopulation, using genes that were upregulated in cells from geographic atrophy cell lines. Using the Disease Ontology database^66^, we identified the pathway associated with AMD was enriched in RPE4 (DOID:10871—*P* value: 5.62 × 10^−6^, *q*-value: 5.00 × 10^−4^). Other disease pathways such as retinal degeneration, diabetic retinopathy and retinal vascular disease, Alzheimer’s disease and tauopathy, vitiligo, metabolism disorders, and various cancers were enriched in other subpopulations (Supplementary Fig. 5). The Gene Ontology database^67^ was used to identify biological processes, cellular components, and molecular functions that may be involved in the pathogenesis of geographic atrophy. We identified pathways related to standard cellular processes such as transcription and translation, protein localization to the endoplasmic reticulum, and metabolism, in addition to pathways related to the mitochondrial metabolic activity such as electron chain transport, oxidative phosphorylation, ATP metabolic process, NAD/NADH metabolic process, mitochondrial respiratory chain complex assembly, and mitochondrial transport (Fig. 2c and Supplementary Data 2). The pathway related to response to reactive oxygen species, was also enriched in many RPE subpopulations (Fig. 2c and Supplementary Data 2).

Altogether, this data points to clear differences in various molecular pathways, in particular associated with mitochondrial activities, metabolic pathways, and regulation of the ECM. Given we retrieved gene expression profiles associated with AMD as observed by others and in other experimental settings, our data clearly confirms that iPSC-derived RPE cells are an adequate model for the study of AMD. This also provides confidence that novel pathways can be uncovered using this in vitro model. At every step, our experimental workflow ensured that both control and geographic atrophy samples were assayed in shared conditions or randomized with respect to disease status (“Methods”). Hence, we are confident these transcriptomic differences are due to the genetic effects underlying the geographic atrophy risk. Moreover, environmental factors are unlikely to account for a difference in gene expression in differentiated cells, given the epigenetic profile of fibroblast-derived iPSCs is reset during reprogramming^68,69^.

### The proteomic analysis of RPE cells confirms specific protein expression in geographic atrophy cells

**Fig. 3:**
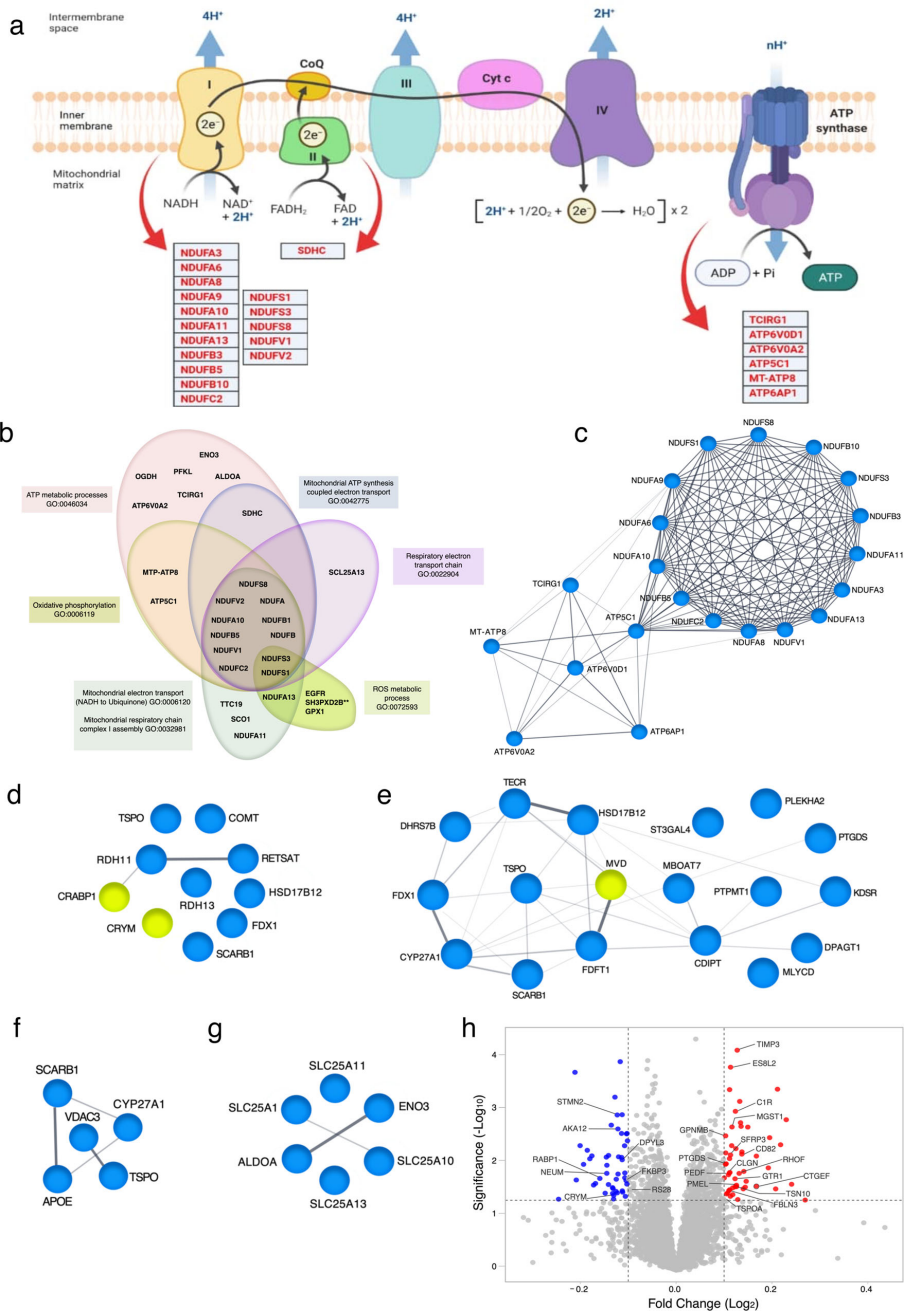
Proteome analysis of control and geographic atrophy RPE cells. **a** Schematic representation of respiratory chain complexes showing proteins that were significantly upregulated in geographic atrophy. Illustration adapted from “Electron Transport Chain”, by BioRender.com (2022). Retrieved from https://app.biorender.com/biorender-templates. **b** Representation of enriched biological processes (Gene Ontology) related to mitochondrial function identified using STRING analysis, yellow circles depict downregulated proteins in geographic atrophy. **c**–**g** Entire local network clustering (STRING) with enriched pathways for oxidative phosphorylation (**c**); hormone metabolic process (**d**); lipid biosynthesis (**e**), cholesterol metabolism (**f**), and gluconeogenesis (**g**). Significantly upregulated proteins in geographic atrophy are highlighted in blue; downregulated depicted in yellow. STRING networks were generated using Biological Process (GO) or KEGG data obtained from analysis of the most significantly up- and downregulated proteins from the proteomic screen (Supplementary Data 1). **h** Annotated volcano plot of the 25 most significantly differentially expressed proteins (based on combined *P* value and fold-change criteria). Upregulated proteins in geographic atrophy are shown in red, and downregulated proteins in blue. Statistical significance established using two-tailed *t* tests. Source data are provided as a Source Data file.

We confirmed the differential gene expression results by measuring protein quantification in all lines using mass spectrometry. From eight TMT experiments, a total of 6923 proteins were identified and quantified at an initial protein FDR of not more than 1% (Supplementary Data 1 and dataset identifier PXD029501 in ProteomeXchange). 234 proteins were identified as differentially abundant between geographic atrophy and control (Student *t* test analysis, *P* < 0.05, fold change ≥1.1, Supplementary Data 1). Given the experimental approach was not based on single cells but on a bulk harvest and analysis for each condition, cell cultures were assessed as a whole without distinction of subpopulations. Similar levels of the canonical RPE markers represented in Supplementary Fig. 3 were detected between the control and geographic atrophy cohorts. This analysis confirmed that specific pathways relating to mitochondrial processes were modulated in geographic atrophy cells (Fig. 3a, b, Supplementary Fig. 6, and Supplementary Data 1). For instance, the increased abundance of many proteins from the respiratory chain pathway in geographic atrophy RPE, suggests increased respiratory activity in geographic atrophy RPE cells. These include *ATP5C1*, *SCO1* and mitochondrial complex I components (*NDUFA3*, *NDUFA6*, *NDUFA8*, *NDUFA9*, *NDUFA10*, *NDUFA11*, *NDUFA13*, *NDUFB3*, *NDUFB5*, *NDUFB10*, *NDUFC2*, *NDUFS1*, *NDUFS3*, *NDUFV1*, *NDUFV2*) (Fig. 3a, c). Other enzymes involved in oxidoreductase activity were also upregulated, such as *DHRS13*, *DHRS7B,* and *GPX1*. Analysis of the proteomics dataset using STRING functional enrichment analysis (Biological Processes—Gene Ontology) supported this observation. The dataset was highly enriched in pathways involved in oxidative phosphorylation, including mitochondrial electron transport (NADH to ubiquinone); mitochondrial respiratory chain complex I assembly; mitochondrial ATP synthesis coupled electron transport; oxidative phosphorylation; ATP metabolism; respiratory electron transport chain and reactive oxygen species (Fig. 3b, Supplementary Fig. 6, and Supplementary Data 1). Many of these same identified proteins were also represented in local network clustering (STRING) analysis (mitochondrial respiratory chain complex; oxidative phosphorylation and proton transporting) (Fig. 3c, Supplementary Fig. 6, and Supplementary Data 1). Markers associated with the pathways identified by GO and STRING analysis were upregulated in the geographic atrophy diseased cohort. Unsurprisingly, KEGG analysis found that oxidative phosphorylation was the most enriched biological process (ranked by strength parameters) in the diseased cohort, with many of the pathways’ hits closely related to the neurodegenerative diseases Parkinson’s and Alzheimer’s (Supplementary Data 1). Further, various metabolic pathways were upregulated in the geographic atrophy cohort, including for the retinoid metabolism (*RETSAT*, *RDH11*, *RDH13*, Fig. 3d), and reduced expression of the retinoic acid-binding protein *CRABP1* - the most decreased protein in the geographic atrophy cohort); lipid including cholesterol metabolism (*MLYCD*, *CYP20A1*, *CYP27A1*, *ACOT1*, *HSD17B12*, *TECR*, *KDSR*, *APOE*, *DHRS13*, Fig. 3e, f), and gluconeogenesis and glycolysis (*ALDOA*, *ENO3*, Fig. 3g). Many proteins involved in cell adhesion and ECM regulation were also upregulated in the geographic atrophy cohort (Supplementary Data 1). These include *TIMP3* (the fifth-highest most abundant protein in the geographic atrophy cohort, Fig. 3h), *EFEMP1*, *ITGB4*, *SERPINB9*; various tetraspanins (*TSPAN6, TSPAN10, CD9/TSPAN29, CD82/TSPAN27*, Fig. 3h), *C1QTNF5* and *BSG*. In the geographic atrophy RPE cells, the proteomic analysis also revealed increased levels of proteins known to be present in drusen^70^, such as *APOE* and *SCARB1*—also involved in cholesterol metabolism—and *TIMP3*. Finally, a number of signaling molecules were also upregulated in the geographic atrophy RPE cells, with increased levels of the growth factor *SERPINF1*/ *PEDF* (Fig. 3h); WNT signaling ligands *SFRP1* and *SFRP3/FRZB* (Fig. 3h); the prostaglandin-synthesizing enzyme *PTGDS* (Fig. 3h). The complement pathway component *C1R* was also highly upregulated in the geographic atrophy RPE cells (Fig. 3h), as observed in other retinal dystrophies’ RPE cells^30^. These data suggest that autocrine/paracrine signaling mechanisms are modified in the geographic atrophy RPE cells. Many of the aforementioned proteins were among the 25 most significantly differentially expressed proteins (Fig. 3h). Altogether, the large-scale proteomics analysis confirmed that proteins and pathways associated with mitochondrial and metabolite functions (including lipid synthesis, gluconeogenesis, cholesterol, and glucose metabolism) are modified in geographic atrophy RPE cells.

### Profiling genetic regulation associated with geographic atrophy in the RPE transcriptome and proteome

We explored the genetic regulation associated with geographic atrophy within each RPE subpopulation by integrating the genotype profiles of each donor with transcriptomic and proteomic data and mapped quantitative trait loci associated with expression (eQTL) and protein (pQTL) levels. Using expression data, we identified 445 cis-eQTL across all subpopulations, which surpassed the study-wide significance of Benjamini–Hochberg FDR < 0.05 and a homozygous alternate allele in at least five individuals (Table 3 and full results in Supplementary Data 3). Only 7.9% of *cis*-eQTL were replicated in more than one subpopulation and the direction of effect was the same in all subpopulations that had the eQTL (Supplementary Fig. 8A and Supplementary Table 7). The majority of genes with an eQTL (eGenes) (62.9%) were subpopulation-exclusive, and only two eGenes—*GSTT1* and *RPS26*—were common to all subpopulations. *RPS26*, which encodes for proteins forming the small subunits of ribosomes, was found to be ubiquitous in iPSC-derived retinal organoids^44^ and has been associated with type 1 diabetes^71^. Two eQTL associated with this gene—rs1131017^27^ from RPE3, and rs10876864^25^ from RPE1 and RPE2 have been previously characterized in bulk studies. *GSTT1* encodes for a glutathione S transferase which is protective against oxidative stress, and has also been associated with disease, including ophthalmic conditions such as glaucoma^72^, diabetic retinopathy^73^, and cataract^74^. Implications of *GSTT1* variants in AMD remain controversial^75,76^. The majority of *cis*-eQTL were detected in the two largest subpopulations RPE1 and RPE2 (78%), and share the greatest number of eQTL (30) (Supplementary Table 8). The effect sizes of these shared *cis*-eQTL are highly correlated (*r* = 0.99, *P* value < 2.2 × 10^−16^) and suggests these variants are common genetic regulation mechanisms in these two subpopulations. It should also be noted that these subpopulations have a less mature RPE expression profile in comparison to other RPE subpopulations.

**Table 3:** Summary of lead *cis*-eQTL per subpopulation.

Subpopulation	Number of eQTLs	Number of eSNPs	Number of interactions
Progenitor RPE	36	36	0
RPE1	230	218	17
RPE2	118	117	9
RPE3	38	37	9
RPE4	4	4	0
RPE5	23	23	3
RPE6	6	6	0
Total	445	391	38

Lead eQTL identified in the preliminary round of mapping then underwent additional testing to identify genotype by disease interactions, and revealed 38 eQTL with significant interactions between these terms (*P* value < 0.05) in RPE1, RPE2, RPE3, and RPE5 (Supplementary Data 3). All eQTL with significant interactions were subpopulation-specific. Interestingly, although the eQTL shared between RPE1 and RPE2 had highly correlated effect sizes in the initial analysis, the interaction effect sizes were not correlated (*r* = 0.64, *P* value = 1.39 × 10^−4^). This may be due to RPE1 having more significant interactions than RPE2. Five of the eGenes were previously detected in the differential expression analysis—*KIFC1* (RPE1), *RPS26* (RPE1, RPE3), *SPATA20* (RPE1, RPE2), *ABR* (RPE1), and *GSTT1* (RPE3). Only three of the eQTL associated with these genes had the same directionality in effect as the fold change in DE analysis, which may be due to the eQTL analysis being based off pseudobulk expression values instead of single-cell expression values.

**Fig. 4:**
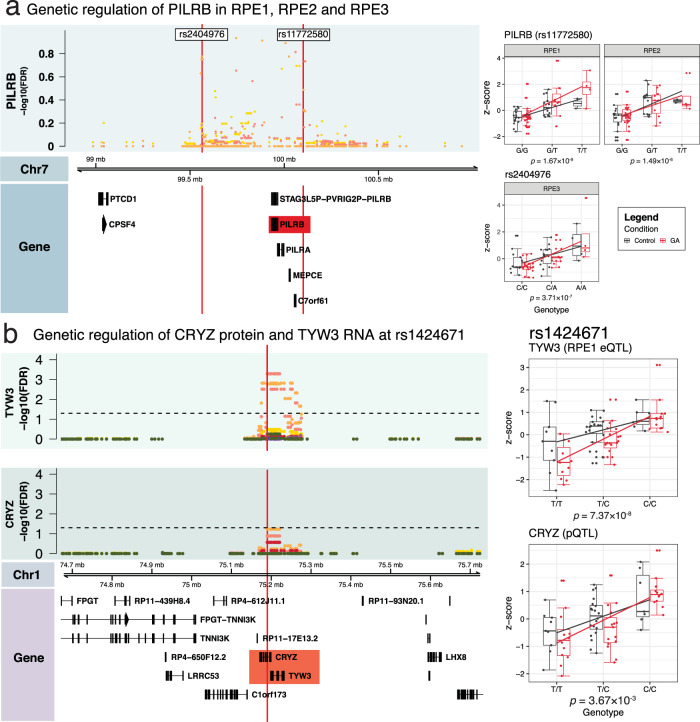
Genetic regulation of disease-associated expression and protein in RPE. Locus zoom plots depict a 500kbp region around SNPs of interest. Scatter plot axes show −log10(FDR) values of eQTL results. Scatter plot colors correspond to the RPE subpopulation the scores come from. Boxplots show aggregated z-scores of individuals, separated by condition and genotype. All boxplots show *P* values for their respective tests (eQTL or pQTL), medians (horizontal line within box), first (25th percentile) and third (75th percentile) quartiles (lower and upper hinges), 1.5 ×  the interquartile range (whiskers) and z-scores of individual samples as points. GA geographic atrophy. **a** PILRB eQTL results at rs11772580 (eQTL in RPE1 and RPE2) and rs2404976 (eQTL in RPE3). n_RPE1_: (control: 35 individuals, GA: 43 individuals), n_RPE2_ = (control: 35 individuals, GA: 43 individuals), n_RPE3_ = (control: 31 individuals, GA: 38 individuals). **b** eQTL and pQTL at rs1424671 that includes TYW3 (eQTL interaction in RPE1) and CRYZ (pQTL interaction in bulk samples). n_RPE1_ = (control: 35 individuals, GA: 43 individuals) individuals, n_Proteomics_ = (control: 36 individuals, GA: 39 individuals).

We reviewed previous studies performed on bulk samples of the retina and RPE/choroid to determine if we detected eQTL related to AMD and geographic atrophy and found 19 eQTL, 213 eGenes and 20 SNPs with the most significant association (eSNPs), have been characterized previously (Supplementary Data 3). Of particular interest was *PILRB* in RPE1 (rs11772580), RPE2 (rs11772580) and RPE3 (rs2404976)^26^, which is located in the *PILRB*/*PILRA* AMD locus^18^ (Fig. 4a). This gene was identified as a putative causal gene for disease associations in a study in ref. ^26^ using colocalization of GWAS and eQTL results. Other studies have shown that an upregulation of this gene was associated with AMD risk^25,27^; we observed this as an increase in the transcription within RPE1 and RPE3. However, the interaction between genotype and disease did not reach significance in any of the subpopulations. The previously characterized variant associated with *PILRA*—rs7803454^18^, did reach study-wide significance in RPE1, RPE2, and RPE6 (Benjamin–Hochberg FDR—RPE1: 2.64 × 10^−6^, RPE2: 9.75 × 10^−4^, RPE6: 2.35 × 10^−2^) and was associated with *PILRB*, but only three individuals in the study had the risk allele. Loci associated with *HTRA1-ARMS2* (rs10490924 & rs11200638) did not reach study-wide significance. We identified an eQTL associated with *CD160* in RPE2 (rs10910829) which also had a significant interaction between disease and genotype; whilst this gene is associated with macular degeneration—it is primarily involved in neovascular disease^77^. We mapped *cis*-eQTL linked to fives genes from the HLA region—*HLA-F*, *HLA-DQB1*, *HLA-B*, and *HLA-C*, and only *HLA-DQB1* had a significant interaction between disease status and genotype in RPE1 (rs17884945, *P* value: 3.34 × 10^−5^) and RPE2 (rs2857209, *P* value: 2.37 × 10^−3^). The variants that were previously linked to increased AMD risk, rs9274390 and rs41563814^78^, were not present in our dataset.

We corroborated information gained from previous proteomic and transcriptomic analysis, and literature to identify eGenes that may be associated with known and potential new mechanisms of disease. The eQTL associated with *ANK3* and rs4948258 in RPE3 had a significant interaction between genotype and disease which decreased expression levels in geographic atrophy cell lines (Supplementary Data 3 and Supplementary Fig. 8). This gene is a member of the *ankyrin* family that acts as adaptor proteins for linking membrane proteins to the underlying cytoskeleton^79^, and variants associated with this gene have previously been linked to psychiatric disorders such as bipolar disorder^80^. *DPYSL4* in RPE1 (rs56307927) and RPE3 (rs2818409) were other eQTL where genotype had a significant interaction with disease, resulting in increased expression of this transcript in geographic atrophy cell lines (Supplementary Data 3 and Supplementary Fig. 8). This gene is suspected to play a role in energy metabolism in adipocytes and cancer cells^81^. *MTG2* in RPE1 (rs2184161) is another gene that plays a role in energy metabolism by its involvement in the formation of mitochondrial ribosomes^82^. *GSDMD* in RPE1 (rs7835865) plays a role in pyroptosis^83^, is highly polymorphic, and variants in structurally important regions have been found to alter the process^84^. *FLVCR1-AS1* in RPE1 and RPE2 (rs2279692) is related to the heme transporter FCVCR1 and is associated with retinitis pigmentosa and other retinal dystrophies^85–87^.

As protein expression does not necessarily correlate well with mRNA expression levels^88^, we performed *cis*-protein quantitative trait loci (*cis*-pQTL) to identify genetic variants that regulate protein expression in the RPE cells in the context of disease. It must, however, be noted that the proteomic analysis was performed on bulk RPE cultures, hence the identified pQTLs cannot be assigned to individual cells or subpopulations. We did not find any significant associations after correction for multiple testing, so we narrowed our search space to lead variants mapped by pQTL analysis that were also found to have significant associations in *cis*-eQTL analysis. From these results, we identified five proteins that share a lead SNP with RPE subpopulation-level eQTLs and three of these—*PYROXD2* (rs942813), *SPATA20* (rs989128), and *CRYZ* (rs1424671) were significant (Benjamini–Hochberg FDR < 0.1) (Table 4, full results in Supplementary Data 3). *CRYZ* (crystallin zeta, also known as quinone reductase) has previously been associated with a pQTL (rs61790703) in the human liver^89^, and is an evolutionarily conserved protein induced under oxidative stress conditions^90^ as well as detoxification of lipid peroxidation products^91^. The lead SNP of this pQTL—rs1424671, is also the lead SNP for *TYW3* in RPE1, and *CRYZ* in RPE2 in eQTL analysis (Fig. 4b). Studies have linked variants in this loci to circulating resistin levels^92^ and risk for amyotrophic lateral sclerosis in the Chinese Han population^93^. *PYROXD2* (pyridine nucleotide-disulphide oxidoreductase domain 2) is a mitochondrial oxidoreductase regulating mitochondrial function and mitochondrial DNA copy number^94^, and has previously been flagged in a GWAS study of vitamin B6 metabolite levels^95^. The function of *SPATA20* (Spermatogenesis Associated 20)’s functions remain elusive. Although pQTL have been identified for some human tissues^96^, these data are —to our knowledge—the first description of RPE-specific pQTL in the retina associated with age-related macular degeneration.

**Table 4:** Variants associated with pQTL and eQTL.

Uniprot ID	rsID	Gene (pQTL)	Subpop	Gene (eQTL)	SNP Chr	SNP Pos	*P* value (pQTL)	Effect size (pQTL)
Q8TB22.2	rs989128	SPATA20	RPE1	SPATA20	17	48636534	1.59E-02	−6.64E-01
Q8N2H3	rs942813	PYROXD2	RPE1	PYROXD2	10	100153608	1.59E-03	6.37E-01
Q08257	rs1424671	CRYZ	RPE1	TYW3	1	75189847	3.66E-03	5.97E-01
Q08257	rs1424671	CRYZ	RPE2	CRYZ	1	75189847	3.66E-03	5.97E-01

### Transcriptome-wide association study analysis identifies novel genetic associations for geographic atrophy

**Fig. 5:**
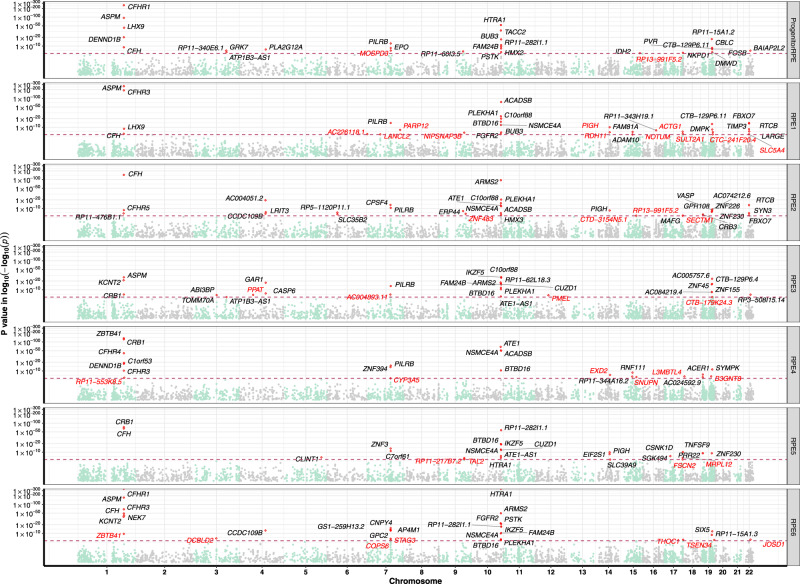
Prioritization of geographic atrophy risk genes. Genes that are significant after Bonferroni correction are highlighted with red dots, with the nearest gene names in black text (previously implicated genes), of which not genome-wide significant in the per-SNP analysis (top GWAS SNP *P* value < 5 × 10^−8^) are highlighted in red text (novel genes identified). The *x* axis is the genome position from chromosome 1 to chromosome 22, the *y* axis is the TWAS *P* value in log-log scale. The maroon horizontal dash line is the Bonferroni correction level. Evidence for association was computed as a Z-score, which was then converted to a two-sided *P* value. *P* values were then adjusted for multiple testing using the Bonferroni method (approximately 19,000 genes in each subpopulation).

We used the iPSC-RPE single-cell eQTL data in conjunction with AMD GWAS summary statistics to prioritize AMD risk genes in a transcriptome-wide association study analysis (TWAS). In the single-cell TWAS, we identified 200 genes associated with AMD after Bonferroni correction in each RPE subpopulation, of which 38 were not genome-wide significant in the per-SNP analysis (best GWAS SNP *P* value < 5 × 10^−8^) (Fig. 5 and Supplementary Data 3). Across different subpopulations, the TWAS results were generally consistent for several well-established regions, such as the *CFH* locus in chromosome 1, the *ARMS2/HTRA1* locus in chromosome 10 and *PILRB* in chromosome 7 (Fig. 5 and Supplementary Data 3). The *CFH* gene was significantly associated in all but the RPE3 and RPE4 subpopulations (Fig. 5 and Supplementary Data 3). Compared to a previous TWAS analysis based on bulk eQTL datasets^97^, *PARP12* gene was also replicated in our single-cell TWAS in the RPE1 cell eQTL data (Fig. 5 and Supplementary Data 3). For the previously reported gene *RLBP1*^97^, nearby gene *IDH2* was identified instead. Interestingly, the top GWAS SNP rs2238307 in gene *IDH2* is highly correlated with the top SNP rs3825991 in *PARP12* (*r*^2^ = 0.77) (Supplementary Data 3).

### Respiration is modified in the geographic atrophy cohort

**Fig. 6:**
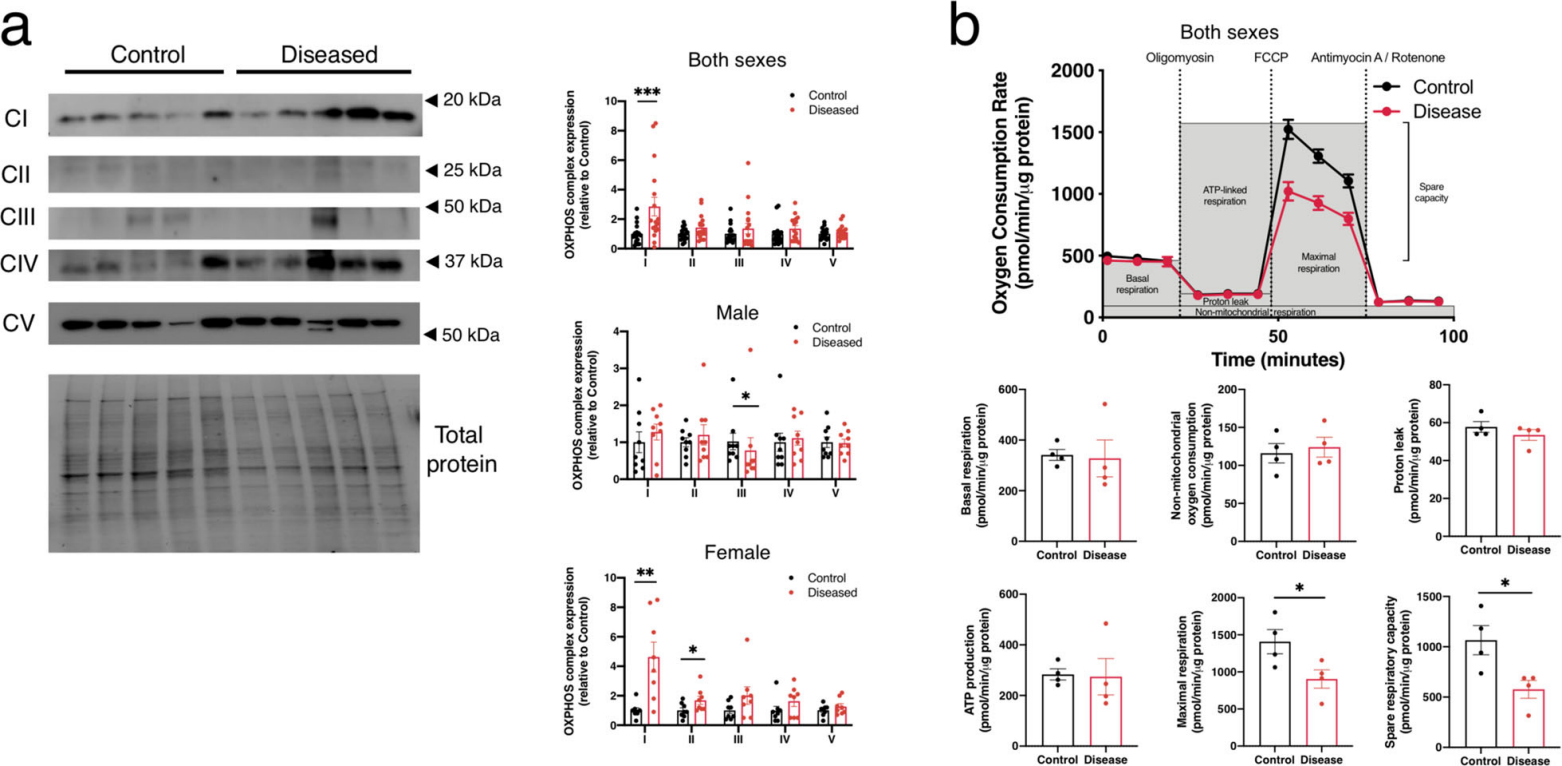
Assessment of mitochondrial function in RPE. **a** Representative immunoblot images of proteins within the mitochondrial electron transport chain, from the control and geographic atrophy female cohorts. Quantification of both sexes indicates an increase in Complex I in the geographic atrophy cohort. Individual male (*n* = 9) and female (*n* = 8) samples show a decrease in Complex III in males and an increase in Complex I and II in females within the geographic atrophy cohort. Original uncropped blots are provided as Supplementary Fig. 9. **b** Oxygen consumption rate was determined using a Seahorse XF24 analyzer. Quantification of basal respiration, non-mitochondrial oxygen consumption, proton leak, ATP production, maximal respiration, and spare respiratory capacity was calculated from four biological samples, containing five technical replicates each. Source data are provided as a Source Data file. **a**—right panels, **b** Data presented are mean +/− SEM, with statistical significance established as *P < 0.05, **P < 0.01, ***P < 0.001, ****P < 0.0001 using two-tailed unpaired *t* tests. (**a**, right panels) both sexes Complex I ****P = 0.007; male Complex III *P = 0.01; female Complex I **P = 0.003; Complex II *P = 0.05. **b** Bottom panels—Seahorse Maximal Respiration. *P = 0.0492; Spare respiratory capacity. *P = 0.0283.

As both transcriptomics and proteomics analyses pointed to clear variations in mitochondrial pathways between the control and disease cohort, we assessed the mitochondria further by performing western blot analysis of mitochondrial electron chain proteins and Seahorse assays of mitochondrial respiratory function in a small number of iPSC-derived RPE cells from control and geographic atrophy. The geographic atrophy cohort showed increased expression of Complex I of the mitochondrial electron transport chain (Fig. 6a and Supplementary Fig. 9). Further analysis of sex-specific changes suggests a decrease in Complex III within males and an increase in Complex I and Complex II in females of geographic atrophy cohort (Fig. 6a and Supplementary Fig. 9). This suggests that mitochondrial complexes are remolded in geographic atrophy, further supporting the observations seen in large-scale proteomics analysis. To investigate the metabolic profile of RPE cells, oxygen consumption rate (OCR) was measured under basal conditions, following oligomycin administration to assess ATP-linked respiration and proton leak, following introduction of an uncoupling agent to induce maximal respiration (carbonyl cyanide 4- (trifluoromethoxy) phenylhydrazone; FCCP), and after rotenone/antimycin A administration, which inhibit Complex I and Complex III respectively and allows for the calculation of non-mitochondrial respiration (Fig. 6b and Supplementary FIg. 9). While basal respiration, mitochondrial ATP production, and proton leak were not different between groups, the geographic atrophy cohort displayed a generalized reduction in both FCCP-stimulated maximal respiration and spare respiratory capacity. Together, the geographic atrophy cohort showed remodeling of mitochondrial protein complexes and mitochondrial respiration capacity.

## Discussion

Here, we present a large-scale scRNA-seq and proteomic analysis of iPSC-derived RPE cells affected in geographic atrophy, which highlights variations in key molecular pathways in the geographic atrophy cohort. It must be noted that the relative immaturity of pluripotent stem cell derivatives is a common feature across differentiation protocols of all lineages, with derivatives often considered to show a closer resemblance to fetal-like cells than adult counterparts^98^. Our transcriptomic data indicates that the RPE cells in culture are on a continuum of maturation, with RPE1 and RPE2 being transcriptionally less mature than the RPE3-6 cells. Yet the bulk cultures demonstrate RPE morphological and functional characteristics as consistently described in many studies^46,99,100^, and validates them as a suited model for investigation of RPE biology in vitro. However, it also highlights the need to take the relatively low transcriptomic maturity into consideration when interpreting data and assessing potential therapeutic targets.

Genes with common risk alleles associated with geographic atrophy and neovascular AMD were upregulated in geographic atrophy RPE cells, including *CFH*, *HTRA1*, *EFEMP1,* and *APOE*, which provide further evidence of their involvement in the pathogenesis of geographic atrophy. Key mitochondrial transcripts and proteins were altered in geographic atrophy RPE cells, with an increased expression of genes of the mitochondrial OXPHOS Complex I machinery, oxidative phosphorylation, mitochondrial respiratory chain complex assembly and mitochondrial transport. Various metabolic pathways were also upregulated in the geographic atrophy cohort, in particular the ATP and the NAD/ NADH metabolic processes. The upregulation of genes and proteins involved in the response to reactive oxygen species in the geographic atrophy RPE cells further supports a key role of cellular stress underlying RPE dysfunctions associated with geographic atrophy progression. The retina is amongst the body’s most metabolically active tissues, and our results suggest perturbations to metabolic homeostasis is a feature of geographic atrophy. Indeed, mitochondrial pathways have been hypothesized to play a role in AMD^36^, and provide potential targets for therapy^32,101^. Another nod to metabolic perturbation in macular degeneration is the long-standing hypothesis that chronic inflammation associated with the disease potentially disturbs the metabolic processes that occur between the RPE and the photoreceptors, leading to increased subretinal lactate concentrations, glycolysis deficit, and increased reactive oxygen species production^102^.

The upregulation of genes and proteins involved in ECM reorganization in the geographic atrophy samples corroborates current knowledge suggesting a role of the interaction of the RPE with the ECM in the development of geographic atrophy. For instance, mutations in *TIMP3* are causative of the macular dystrophies Sorsby fundus dystrophy and in *EFEMP1 of* Doyne honeycomb retinal dystrophy and Malatia Leventiese, which are characterized by drusen accumulation underneath the RPE, an aspect that has been recapitulated in vitro using patient iPSC-derived RPE cells^30^. Indeed, proteomic studies on drusen composition have identified TIMP3 and APOE as common constituents^103^, both of which had their expression increased in the geographic atrophy cohort. Similarly, variants in *C1QTNF5*, a membrane protein involved in cell adhesion and secretion, are associated with late-onset retinal degeneration^104–107^.

Retinoid and pigmentation-related proteins were amongst the most significantly differentially expressed between the control and geographic atrophy samples. This includes the retinoid-binding protein CRABP1, downregulated in the geographic atrophy cohort, which complements data showing decreased measurements of CRABP1 in late stage AMD eyes^108^. Taken together, our data suggest that RPE cells from the geographic atrophy cohort show constitutive differences in visual cycle processes and melanosome function to healthy RPE cells. Whether these differences are simply associated or causative of phenotypes remains to be assessed.

Traditionally, eQTL studies are performed on bulk tissues using RNA-seq, so previous AMD studies are based on the retina^25–27^, the choroid, and RPE^26^. We chose scRNA-seq because we wanted to determine if this platform is capable of studying eQTL in the context of cell subpopulations, and if these results recapitulated results from bulk eQTL studies. The majority of eQTL we identified were cell subpopulation-specific, even though the bulk culture was phenotypically RPE. Our study was able to identify eQTL that were characterized previously in bulk studies, which demonstrates iPSC-RPE generated from geographic atrophy patients are capable of replicating genetic regulation mechanisms associated with the disease. Our power to detect eQTL was substantially less, however, due to the number of individuals in this study and limitations related to the application of statistical models designed for bulk methods to single-cell data. The introduction of an interaction term between genotype and disease enabled us to refine our list of possible candidates for further study; one candidate is *GSDMD* (gasdermin) in the RPE1, which is a potential target for inflammatory conditions^109^, and gasdermin D is elevated in eyes from geographic atrophy patients where it plays a key role in the NLRP3 inflammasome activation and subsequent RPE death^110^. Another candidate is the antisense long noncoding RNA (lncRNA) *FLVCR1-AS1* which had significant interactions in RPE1 (rs61832055) and RPE2 (rs2279692). This lncRNA has been implicated in the progression of osteosarcoma by activating the wnt/β-catenin pathway^111^, which has been implicated in the mediation of inflammation in AMD^112,113^. The role of antisense RNA in the pathogenesis of AMD has also been investigated in bulk RNA-seq^60^, which demonstrated the transcription of antisense RNA is substantially greater in retina collected from deceased AMD patients. The identification of eQTL in genes encoding mitochondrial proteins and respiratory complexes, such as *MTG2* and *DPYSL4* further suggests that variations in mitochondrial activities in the RPE might be at play in the progression of geographic atrophy.

The investigation of regulatory mechanisms of protein expression by pQTL analysis shed light on five pQTL variant–protein interactions. In particular, *PYROXD2* regulate mitochondrial functions via oxidoreductase activity^94^ or mitochondrial dynamics^114^. *CRYZ* is induced under oxidative stress^90^ and detoxification of lipid peroxidation products^91^, both molecular events already implicated in AMD pathogenesis^115–117^. These variants regulate protein expression and abundance in the RPE cells and thus further highlight the important role of genetic effects on protein expression in geographic atrophy.

The TWAS analysis identified 200 genes associated with AMD, confirming known associations for AMD, including in the *CFH* and *ARMS2/HTRA1* loci, and also identifying 38 novel genes associated with geographic atrophy, including *IDH2* a new potential causal genes for AMD, given its role in mitochondrial biology and in protecting against oxidative damage^118^.

In addition, assessment of the respiratory chain composition and function in the iPSC-RPE cells validates the results obtained from our -omics analysis that mitochondrial functions are modified in geographic atrophy. Indeed, maximal respiration was impaired in RPE diseased cells which coincided with an increase of mitochondrial Complex I. This data also clearly identified mitochondrial proteins as potential targets to prevent or alter the course of geographic atrophy pathogenesis. This adds to the growing evidence of mitochondrial dysfunctions in neurodegenerative disease, including in AMD^32^ or Parkinson’s disease^119^. Finally, our data on a reduced cohort of line suggests sex-based differences on mitochondrial phenotypes in the geographic atrophy cohort. In particular, RPE cells from female individuals of the geographic atrophy cohort showed a higher increase in Complex I formation than males, suggestive of a stronger underlying mitochondrial difference in females than in males. The role of sex-based differences in the pathogenesis of AMD and geographic atrophy are not yet fully elucidated, with studies describing either an association of sex with pathogenesis or not^120^. In this context, our data could hint that such an effect is observed in RPE cells, with a regulation of mitochondrial functions differing between sexes.

In summary, we have identified important constitutive differences in RPE homeostasis associated with geographic atrophy when compared to healthy RPE cells. Although other work recently reported on the transcriptomic and proteomic profiles of 151 independent iPSCs^121^, this work is the first description of a population-scale analysis of the transcriptome and proteome of iPSC-derived RPE cells, as well as associated with geographic atrophy.

## Methods

### Participant recruitment

All participants gave informed written consent. This study was approved by the Human Research Ethics committees of the Royal Victorian Eye and Ear Hospital (11/1031H, 13/1151H-004), University of Melbourne (1545394), University of Tasmania (H0014124) UWA (2021/ET000366) as per the requirements of the NHMRC, in accordance with the Declarations of Helsinki. Cases that had advanced AMD with geographic atrophy in at least one eye and an age at first diagnosis over 50 years, were recruited through local ophthalmic clinics (mean ± SD age at recruitment: 83.8 ± 8.2 years). The control cohort has previously been described in ref. ^44^, and had no manifest ophthalmic disease or drusen. The mean ± SD age at recruitment for participants was 69.8 ± 9.5 years. To ensure a diagnosis of AMD and not a monogenic retinal disease-causing atrophy, all case participants had drusen identified on clinical examination. Dominantly inherited drusen phenotypes such as Sorsby fundus dystrophy, Doyne’s honeycomb dystrophy and Malattia Leventinese as well as fleck dystrophies such as Stargardt’s disease were excluded.

### Fibroblast culture

Punch biopsies were obtained from subjects over the age of 18 years. Fibroblasts were expanded, cultured, and banked in DMEM with high glucose, 10% fetal bovine serum (FBS), l-glutamine, 100 U/mL penicillin, and 100 μg/mL streptomycin (all from Thermo Fisher Scientific, USA). All cell lines were mycoplasma-free (MycoAlert mycoplasma detection kit, Lonza, Switzerland). Fibroblasts at passage (p) 2 were used for reprogramming.

### Generation, selection, and iPSC maintenance

The maintenance and passaging of iPSCs were performed using a TECAN liquid handling platform as described^41^. Briefly, iPSCs were generated by the nucleofection (Lonza) of episomal vectors expressing *OCT-4*, *SOX2*, *KLF4*, *L-MYC, LIN28,* and shRNA against *p53*^122^ in feeder- and serum-free conditions using TeSR™-E7™medium (Stem Cell Technologies) as described^41^. Pluripotent cells were selected using anti-human TRA-1-60 Microbeads (Miltenyi)^43^ and subsequently maintained onto vitronectin XF™-coated plates (Stem Cell Technologies) in StemFlex™ (Thermo Fisher Scientific), with media changes every second day and weekly passaging using ReLeSR™ (Stem Cell Technologies). Pluripotency was assessed by expression of the markers OCT3/4 (sc-5279, dilution 1/40, Santa Cruz Biotechnology) and TRA-1-60 (MA1-023-PE, dilution 1/100, Thermo Fisher Scientific) by immunocytochemistry, and virtual karyotyping by CNV array on all lines, as described in ref. ^41^. Only geographic atrophy lines were generated for this study, as all control lines were already generated, and characterized in ref. ^44^.

### Differentiation of iPSCs into RPE cells

RPE cells were generated as we described previously^42^. Briefly, iPSCs were differentiated in E6 medium (Stem Cell Technologies) containing N2 supplement (Life Technologies), penicillin–streptomycin (Life Technologies) for 21–38 days (to reach RPE differentiation), switched to RPE medium (αMEM, 5% FBS, non-essential amino acids, N1 supplement, penicillin–streptomycin–glutamate, taurine–hydrocortisone–triiodothyronine), and cultured for a further 22–29 days with media changes every 2–3 days. Cells were then passaged with 0.25% trypsin-EDTA and plated onto growth factor-reduced Matrigel-coated plates (Corning) to enrich in RPE cells for an additional 30 days (76–88 days total).

### Immunocytochemistry of RPE cells

Cells fixed with 4% paraformaldehyde (8 min, 4 °C), permeabilized and blocked with a solution of 0.2% v/v Triton X-100 and 5% BSA in PBS (60 min, 4 °C) were incubated with the following primary antibodies (all in PBS—5% BSA, 4 °C, overnight): ZO-1 (#339100, 10 μg/mL, Life Technologies), PMEL (ab137062, 5 μg/mL, Abcam), BESTROPHIN (ab2182, 2 µg/mL, Abcam), OCCLUDIN (33-1500 (OC-3F10), 3 µg/mL, Thermo Fisher Scientific), RPE65 (ab235950, 10 μg/mL, Abcam), washed three times in PBS and incubated with isotype-specific secondary antibodies (Alexa Fluor 568 Goat Anti-Mouse IgG, # A-11031, 1:1000; and Alexa Fluor 488 Goat Anti-Mouse IgG, # A-11029, 1:1000 both from Thermo Fisher Scientific). Nuclei were counterstained using bisBenzimide Hoechst 33342 trihydrochloride, Sigma-Aldrich) and mounted in ProLong™ Gold Antifade Mountant. The specificity of the staining was verified by the absence of staining in negative controls consisting of the appropriate negative control immunoglobulin fraction (Dako). Images were acquired on a Zeiss Axio Imager M2 fluorescent microscope using ZEN Blue 3.5 software (Zeiss).

### Transepithelial electrical resistance (TEER)

TEER measurements were taken under sterile conditions using a EVOM^2^ voltohmmeter (World Precision Instruments) on a heated platform set to 37 °C. TEER measurements were taken from five independent iPSC-derived RPE wells. Net TEER measurements were calculated by subtracting the value of a blank, Matrigel-coated filter without cells from the experimental value. Final resistance-area products (Ω cm^2^) were obtained by multiplying by the growth area of the permeable Transwell insert. Results were graphed using Graph Pad PRISM 9.

### RPE cell harvest and single-cell preparation

RPE cells were harvested with 0.25% Trypsin-EDTA for 8 min, inactivated with RPE medium, and dissociated using manual trituration to yield a single-cell suspension. The cell suspension was centrifuged (5 min, 300 × *g*, 4 °C), following which cells were resuspended in 1 mL of 0.1% BSA in PBS solution. Subsequently, cells were counted and assessed for viability with Trypan Blue, then pooled (eight samples maximum) at a concentration of 1000 live cells/μl (1 × 10^6^ cells/mL).

### Single-cell 3’ RNA-sequencing and pre-processing of transcriptome data

Multi-donor single-cell suspensions were prepared for scRNA-seq using the Chromium Single Cell 3′ Library & Gel bead kit (10x Genomics; PN-120237). Each pool was loaded onto individual wells of 10x Genomics Single Cell A Chip along with the reverse transcription (RT) master mix to generate single-cell gel beads in emulsion (GEMs). The Reverse transcription was performed using a C1000 Touch Thermal Cycler with a Deep Well Reaction Module (Bio-Rad) as follows: 45 min at 53 °C; 5 min at 85 °C; hold 4 °C. cDNA was recovered and purified with DynaBeads MyOne Silane Beads (Thermo Fisher Scientific; catalog no. 37002D). Subsequently, it was amplified as follows: for 3 min at 98 °C; 12× (for 15 s at 98 °C; for 20 s at 67 °C; for 60 s at 72 °C); for 60 s at 72 °C; hold 4 °C followed recommended cycle number based on targeted cell number. Amplified cDNA was purified with SPRIselect beads (Beckman Coulter; catalog no. B23318) and underwent quality control following the manufacturer’s instructions. Sequencing libraries for each pool were labeled with unique sample indices (SI) and combined for sequencing across two 2 ×150 cycle flow cells on an Illumina NovaSeq 6000 (NovaSeq Control Software v1.6) using S4 Reagent kit (catalog no. 20039236). Raw base calls from the sequencer then underwent demultiplexing, quality control, mapping, and quantification with the Cell Ranger Single Cell Software Suite 3.1.0 by 10x Genomics (https://www.10xgenomics.com/). The *count* pipeline was run on each pool, with the target cell number set to 20,000 and demultiplexed reads mapped to the *Homo sapiens* reference *hg19*/*GRCh37* from ENSEMBL (release 75). The resulting transcriptome data for each pool then underwent quality control using the *Seurat* R package^123^. Cells were removed if they did not meet the upper and lower thresholds calculated from 3 Median Absolute Deviations (MAD) of total UMI counts and number of detected genes, and if transcripts from mitochondrial genes exceeded 25% of total transcripts. Raw UMI counts from remaining cells then underwent normalization and scaling using the SCTransform function as implemented in Seurat^124^.

### SNP genotype analysis and imputation

DNA was extracted from cell pellets using QIAamp DNA Mini Kit (QIAGEN, 51306) following the manufacturer’s instructions. DNA concentration was determined with a SimpliNano spectrophotometer (GE Life Sciences), diluted to a final concentration of 10–15 ng/µl, and samples were genotyped on the UK Biobank Axiom™ Arrays at the Ramaciotti Centre for Genomics, Sydney, Australia. Genotype data were exported into PLINK data format using GenomeStudio PLINK Input Report Plug-in v2.1.4 and screened for SNP and individual call rates (<0.97), HWE failure (*P* < 10^−6^), and MAF (<0.01). Samples with excess autosomal heterozygosity or with sex-mismatch were excluded. In addition, a genetic relationship matrix from all the autosomal SNPs were generated using the GCTA tool and one of any pair of individuals with estimated relatedness larger than 0.125 were removed from the analysis. Individuals with non-European ancestry were excluded outside of an “acceptable” box of + /− 6 SD from the European mean in PC1 and PC2 in a SMARTPCA analysis. The 1000 G Phase 3 population was used to define the axes, and the samples were projected onto those axes. Imputation was performed on each autosomal chromosome using the Michigan Imputation Server with the Haplotype Reference Consortium panel (HRC r1.1 2016) and run using Minimac3 and Eagle v2.3^125,126^. Only SNPs with INFO > 0.8 and MAF > 0.1 were retained for downstream analyses.

### Demultiplexing of cell pools into individual donors

We used two SNP-based demultiplexing methods (popscle demuxlet v0.1-beta^127^ and souporcell v2.0^128^) and two transcription-based doublet-detection methods (scrublet v0.2.1^129^ and DoubletDetection v2.5.2) to identify droplets that contained one cell (singlets) and assign those cells to the correct donor. Droplets were considered singlets if they were classified as a singlet by all four softwares and were assigned to the same individual by both demuxlet and souporcell. For demuxlet, allele frequencies were first calculated with *popscle dsc-pileup* using all default parameters and known SNP locations based on imputed SNP genotypes overlapping exons. To classify doublets and assign singlets to each individual we ran *popscle demuxlet* using those pileup counts with default parameters except—geno_error_offset set to 0.1 and—geno_error_coeff set to 0. Souporcell was used to classify droplets as doublets or singlets and to assign the singlets to clusters with *souporcell_pipeline.py* using default parameters and the—common_variants parameter to use just the known SNP locations overlapping exons that had been imputed for the individuals in the study. The SNP genotypes of the identified clusters were then correlated with the reference SNP genotypes. A cluster was assigned to a given individual if the correlation between them was the highest for both that individual and that cluster. Scrublet was run four different times using four different minimum variable percentile gene levels: 80, 85, 90, and 95 with all other default recommendations. The best variable percentile gene level was selected based on the best bimodal distribution of the simulated doublets with a reasonable doublet threshold (i.e., at the lowest point between the bimodal distribution). DoubletDetection detected doublets based on simulated transcriptional profiles by using the *doubletdetection.BoostClassifier* function with 50 iterations, *use_phenograph* set to False and *standard_scaling* set to True. The number of doublets per iteration were visualized to ensure convergence.

### Classification of cell subpopulations

Cells were assigned to RPE subpopulations using a supervised cell classification method called *scPred* v0.9^47^. A classifier was prepared from a reference dataset that had been characterized in a previous study^42^, and consisted of iPSC-derived RPE cells that had been profiled at two time points: 90 and 455 days of differentiation. The 90 day-time point corresponds to the time point chosen for these experiments. Expression data from the reference was normalized using the *SCTransform* method from Seurat v3.1.5^124^, log2-transformed and scaled. The normalized values were then reduced to 15 Principal Components (PCs) with the *eigenDecompose* function from *scPred* and used to train the model with default settings. Classification was performed on each batch, and each cell was assigned a probability of belonging to a reference cluster based on the fit of its expression profile to the reference. To account for the transitional nature of cells from this experiment, we applied an adaptive threshold based on a cell having a prediction probability that lies within 0.3 standard deviations of the mean of all prediction probabilities of a reference subpopulation.

### Integration and dimensionality reduction of transcriptome data

Transcriptome data from all pools were integrated using the reciprocal Principal Component Analysis (RPCA) workflow from Seurat v4.0.5^123^. This workflow was selected due to the large size of the dataset and commonalities between samples that include a shared sequencing platform, identical culture conditions, and cell types. Anchors for integration were selected from 3000 of the most variable genes using RPCA, and pools were aligned using the top 30 PCs and 20 neighbors. The first two pools were used as references for the alignment of the other tools to speed up the integration process. The resulting integrated assay was used to generate shared embeddings with Principal Component Analysis (PCA), and subsequently Uniform Manifold Approximation Projection (UMAP)^130^. Inspection of these projections showed batch effects related to pool and donor were minimal after integration.

### Curation of RPE-specific gene sets and gene signature analysis

Gene sets representing the transcriptomic signatures were drawn from the resources made available in ref. ^50^ for adult RPE. Genes were selected from RPE-specific markers identified via DGE based on significance and size of fold change (*P* value < 0.05, average log2 Fold Change >0.25). Gene signature scores for each cell were then calculated with the UCell^51^ R package.

### Differential expression analysis

Gene markers specific to disease status and subpopulation were identified using differential expression analysis, as implemented in MAST v1.16^131^. MAST was run through Seurat’s FindMarkers function on log-transformed, unnormalized UMI counts with the following latent variables: total UMI count (library size), pool number, age and sex. Sex-specific effects were identified by testing within each subpopulation and comparing results from control and AMD groups. Bonferroni correction was used to correct *P* values for multiple testing, and the threshold for significance was |Average log2 fold change | > 0.25 and adjusted *P* value < 1.8 × 10^−5^. The replication rate was defined by taking the number of genes common to this study and the bulk DE study with the same direction of effect, and dividing it by the number of DE genes from this study.

### Gene ontology and disease ontology over-representation analysis

Gene sets were prepared for each subpopulation, from genes identified via standard differential expression analysis. If fold-change information was available, genes were also grouped by direction of regulation. Over-representation analysis was performed with the Gene Ontology^67,132^ and Disease Ontology^133^ databases, as accessed through *clusterProfiler*^65^. *P* values were corrected for multiple testing using Benjamini & Hochberg FDR and filtered for significance using a *q*-value threshold of 0.2.

### Transcriptome-wide association study

TWAS was implemented in the FUSION pipeline 2019/10/01 (available at https://github.com/gusevlab/fusion_twas)^134^. We first computed the single-cell eQTL weights using the “blup”, “lasso”, and “enet” models^134^ in each subpopulation. Then, the single-cell gene-expressions were used in a TWAS using AMD GWAS summary statistics^21^ to evaluate the association between inferred gene expression and AMD. Evidence for association was computed as a Z-score, which was then converted to a two-sided *P* value. *P* values were then adjusted for multiple testing using the Bonferroni method (~19,000 genes in each subpopulation).

### Preparation of protein samples

RPE cell cultures were lysed in RIPA buffer supplemented with phosphatase and protease inhibitors, sonicated with a probe sonicator (40 HZ × 2 pulses × 15 s), and insoluble debris were removed by centrifugation at 14,000 rpm for 15 min at 4 °C, prior to measurement of protein contents by standard bicinchoninic acid assay (MicroBCA protein assay kit, Thermo Scientific). Solubilised proteins were reduced using 5 mM dithiothreitol and alkylated using 10 mM iodoacetamide. Proteins (150 µg) were initially digested at room temperature overnight using a 1:100 enzyme‐to‐protein ratio using Lys‐C (Wako, Japan), followed by digestion with Trypsin (Promega, Madison, WI) for at least 4 hours at 37 °C also at a 1:100 enzyme‐to‐protein ratio. Resultant peptides were acidified with 1% trifluoroacetic acid and purified using styrene divinylbenzene‐reverse-phase sulfonate (Empore) stage tips. The proteome was identified on a Tandem Mass Tag (TMT) platform (Progenetech, Sydney, Australia).

### TMT labeling

Eight independent 10 plex TMT experiments were carried out. Briefly, dried peptides from each sample were resuspended in 100 mM HEPES (pH 8.2) buffer and peptide concentration measured using the MicroBCA protein assay kit. Sixty micrograms of peptides from each sample was subjected to TMT labeling with 0.8 mg of reagent per tube. Labeling was carried out at room temperature for 1 h with continuous vortexing. To quench any remaining TMT reagent and reverse the tyrosine labeling, 8 μl of 5% hydroxylamine was added to each tube, followed by vortexing and incubation for 15 min at room temperature. For each of the respective ten plex experiments, the ten labeled samples were combined, and then dried down by vacuum centrifugation. Prior to High-pH reversed-phase fractionation, the digested and TMT-labeled peptide samples were cleaned using a reverse-phase C18 clean-up column (Sep-pak, Waters) and dried in vacuum centrifuge. The peptide mixture was resuspended in loading buffer (5 mM ammonia solution (pH 10.5), separated into a total of 96 fractions using an Agilent 1260 HPLC system equipped with a quaternary pump, a degasser and a Multi-Wavelength Detector (MWD) (set at 210-, 214-, and 280-nm wavelength). Peptides were separated on a 55-min linear gradient from 3 to 30% acetonitrile in 5 mM ammonia solution pH 10.5 at a flow rate of 0.3 mL/min on an Agilent 300 Extend C18 column (3.5-μm particles, 2.1 mm ID and 150 mm in length). The 96 fractions were finally consolidated into eight fractions. Each peptide fraction was dried by vacuum centrifugation, resuspended in 1% formic acid, and desalted again using SDB-RPS (3M-Empore) stage tips.

### Liquid chromatography-electrospray ionization tandem mass spectrometry (LC-ESI-MS/MS) data acquisition

Mass spectrometric data were collected on an Orbitrap Lumos mass spectrometer coupled to a Proxeon NanoLC-1200 UHPLC. The 100-µm capillary column was packed with 35 cm of Accucore 150 resin (2.6 μm, 150 Å; Thermo Fisher Scientific). The scan sequence began with an MS1 spectrum (Orbitrap analysis, resolution 60,000, 400–1600 Th, automatic gain control (AGC) target 4 × 10^5^, maximum injection time 50 ms). Data were acquired for 90 min per fraction. Analysis at the MS2 stage consisted of higher energy collision-induced dissociation (HCD), Orbitrap analysis with the resolution of 50,000, automatic gain control (AGC) 1.25 ×105, NCE (normalized collision energy) 37, maximum injection time 120 ms, and an isolation window at 0.5 Th. For data acquisition including FAIMS, the dispersion voltage (DV) was set at 5000 V, the compensation voltages (CVs) were set at −40 V, −60 V, and −80 V, and TopSpeed parameter was set at 1.5 s per CV.

### Proteomic data analysis

Spectra were converted to mzXML via MSconvert v3.0. Database searching included all entries from the Human UniProt Database (downloaded: August 2019). The database was concatenated with one composed of all protein sequences for that database in the reversed order. Searches were performed using a 50-ppm precursor ion tolerance for total protein-level profiling. The product ion tolerance was set to 0.2 Da. These wide mass tolerance windows were chosen to maximize sensitivity in conjunction with Comet searches and linear discriminant analysis. TMT tags on lysine residues and peptide N-termini (+229.163 Da for TMT) and carbamidomethylation of cysteine residues (+57.021 Da) were set as static modifications, while oxidation of methionine residues (+15.995 Da) was set as a variable modification. Peptide-spectrum matches (PSMs) were adjusted to a 1% false discovery rate (FDR). PSM filtering was performed using a linear discriminant analysis, as described previously and then assembled further to a final protein-level FDR of 1%, using the Picked FDR method^135^. An isolation purity of at least 0.7 (70%) in the MS1 isolation window was used for samples. For each protein, the filtered peptide TMT SN values were summed to create protein quantifications. To control for different total protein loading within a TMT experiment, the summed protein quantities of each channel were adjusted to be equal within the experiment. Proteins were quantified by summing reporter ion counts across all matching PSMs, also as described previously^136^. Reporter ion intensities were adjusted to correct for the isotopic impurities of the different TMT reagents according to manufacturer specifications. Finally, each protein abundance measurement was scaled, such that the summed signal-to-noise for that protein across all channels equaled 100, thereby generating a relative abundance (RA) measurement. Investigation of protein–protein interactions and functional enrichment GO analysis of DE proteins were performed with the online STRING database version 11.0. STRING analysis^137^ on the entire proteomics dataset, generating a network of interactions (based on both evidence of functional and physical interactions). The interaction score of 0.7 was considered for the visualization of the networks. Network lines represent the protein interaction score, which was set at a minimum medium confidence (0.4). Active interaction sources were based on text mining, experiments, databases, co-expression, neighborhood, gene fusion, and co-occurrence data. Two criteria were applied to determine significantly regulated proteins: fold change over 1.1 and *P* value lower than 0.05.

### Mapping of expression and protein QTL

QTL mapping was performed using expression and protein measurements, and the genotype data of cell line donors that had been filtered for common SNPs (4,309,001 SNPs, Minor Allele Frequency >10%). For eQTL, a donor-gene matrix was generated for each RPE subpopulation by taking the mean of normalized, corrected UMI counts for each gene from cells belonging to each donor that was present in the subpopulation. Genes that were expressed in less than 30% of the donor population were excluded. The resulting values were log-transformed and quantile normalized with the “normalizeBetweenArrays” function from the limma R package^138^. MatrixEQTL was run with an additive linear model using sex, age, disease status, and the top six genotype principal components as covariates, and lead eQTL was selected based on the following thresholds: FDR (Benjamini–Hochberg procedure) <0.05 and homozygous alternate allele frequency >5. To identify eQTL that had alternative allelic effects under different disease statuses, we included an interaction term (SNP: disease status) in the original linear model for each eQTL identified by preliminary mapping and filtered by *P* value < 0.05 of the interaction term. pQTL mapping was performed at a bulk level using protein abundance measurements taken from individual RPE cell lines. Abundance measurements were normalized using rank-based inverse normal transformation, and the protein-donor matrix was converted to an exon-donor matrix by matching protein identifiers and isoforms to exons listed in the Ensembl *Homo sapiens* Genes database^139,140^. SNPs that were within 1MB of exons were selected for mapping, which was performed with the *cis* function from QTLtools^141^. As the same protein measurement was used for each exon belonging to a protein, the abundance measurements for each exon were grouped by protein and the mean value of each group - the measurement of each protein, was used for testing. pQTL results were matched to eQTL results by matching lead SNPs from pQTL analysis to SNPs with an eGene from *cis*-eQTL analysis. Benjamini & Hochberg FDR values for filtered pQTL results were calculated using adjusted beta *P* values and filtered for significance using a threshold of 0.1.

### Western blotting

Proteins lysates obtained for the mass spectrometry analysis were also used for western blots and were normalized via sample protein concentration. Proteins were separated by SDS-PAGE on a 10% acrylamide gel (100 V, 90 min) and transferred onto PVDF membranes (50 V, 90 min). Membranes were blocked with 5% skim milk powder and probed with antibodies against Total OXPHOS (MS604-300, 10 μg/mL, Abcam) or specific IgG isotype control (Invitrogen). Immunoreactive bands were detected with HRP-conjugated sheep anti-mouse IgG antibody (NA9310V, GE Healthcare, UK Limited, 1:10,000) and visualized with the Pierce ECL Western blotting substrate (Thermo Scientific), according to the manufacturer’s instructions. Stain-free images were collected after transfer to correct for loading differences across samples (ChemiDoc MP and ImageLab software Version 4.1, Bio-rad Laboratories, NSW, Australia). Analysis was performed on 17 cell lines on two independent experiments. Results are presented as the density of the immunoreactive band relative to total protein loading for that specific lane (Fiji/ImageJ software Version 2.0), are presented as mean ± SEM and were graphed using Graph Pad PRISM v9. Statistical significance was established as *P < 0.05, **P < 0.01, ***P < 0.001, ****P < 0.0001 using two-tailed unpaired *t* tests.

### Assessment of mitochondrial functions

Basal respiration, ATP-linked respiration, maximal and reserve capacities, and non-mitochondrial respiration of RPE cells were assessed using a Seahorse XF Cell Mito Stress Test Kit (Agilent) and measured with Seahorse XFe24 Analyzer (Agilent). Briefly, cells were cultured in microplates, in prewarmed Seahorse XF assay medium (Seahorse Bioscience) (pH 7.4) supplemented with 25 mM glucose, 1 mM sodium pyruvate and 1 mM glutamine and placed in an incubator at 37 °C for 60 min before experiments. After baseline measurement (i.e., basal), oligomycin, FCCP (carbonyl cyanide 4- (trifluoromethoxy) phenylhydrazone), and rotenone/antimycin A were sequentially injected into each well at 2 µM, 1.5 µM, and 1.5 µM/1.5 µM, respectively. The Seahorse Analyzer was run using 9-min cyclic protocol commands (mix for 3 min, stand for 3 min, and measure for 3 min) in triplicate. Real-time oxygen consumption rate (OCR) assay was performed on 4 cell lines in two independent experiments, with five technical replicates. Results are presented as mean ± SEM and were graphed using Graph Pad PRISM v9. Statistical significance was established as *P < 0.05, **P < 0.01, ***P < 0.001, ****P < 0.0001 using two-tailed unpaired *t* tests.

### Reporting summary

Further information on research design is available in the Nature Research Reporting Summary linked to this article.

## Supplementary information

Supplementary Information

Description of Additional Supplementary Files

Supplementary Data 1

Supplementary Data 2

Supplementary Data 3

Reporting Summary

## Data Availability

All sequencing and processed transcriptomic data generated in this study have been deposited in the ArrayExpress database under accession identifier E-MTAB-11642, and the mass spectrometry raw file and search results have been deposited to the ProteomeXchange Consortium via the PRIDE^142^ partner repository with the dataset identifier PXD029501 (http://www.ebi.ac.uk/pride). Source data are provided with this paper.

## Source data

Source Data
