# Supplementary material for: Transcriptomic and proteomic retinal pigment epithelium signatures of age-related macular degeneration
Source: Nat Commun. 2022 Jul 26;13:4233. doi: 10.1038/s41467-022-31707-4 (PMC9325891; doi:10.1038/s41467-022-31707-4)
Supplement: Supplementary file 1 — Supplementary Information [file 41467_2022_31707_MOESM1_ESM.pdf]

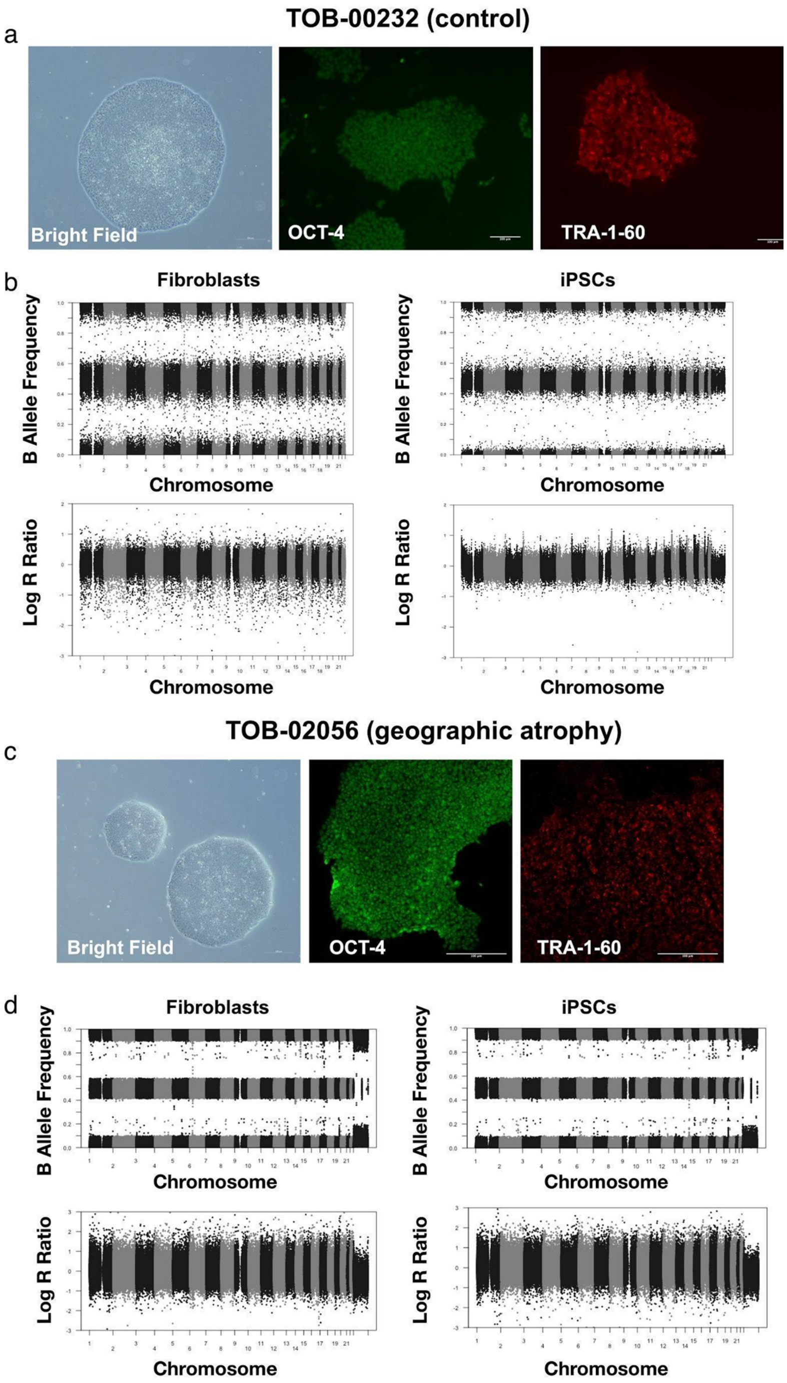

Supplementary Figure 1. Examples of quality control for iPSC lines, Related to Figure 1. Control line TOB-00232 (**a, b**) and geographic atrophy line TOB-02056 (**c, d**) are shown. (**a, c**) Representative bright field images and immunostainings for the pluripotent markers OCT-4 and TRA-1-60 for both lines (Scale bars: 200 µm for bright field, and 100 µm for immunofluorescence). Images are representative of all cell lines. (**b, d**) Copy Number Variation Analysis for both parental fibroblasts and iPSCs representative of a normal virtual karyotyping. Each panel shows the B allele frequency (BAF) and the log R ratio (LRR). BAF at values other than 0, 0.5 or 1 indicate an abnormal copy number. LRR represents a logged ratio of "observed probe intensity to expected intensity". A deviation from zero corresponds to a change in copy number. Quality control was performed on all lines (images and copy number variation).

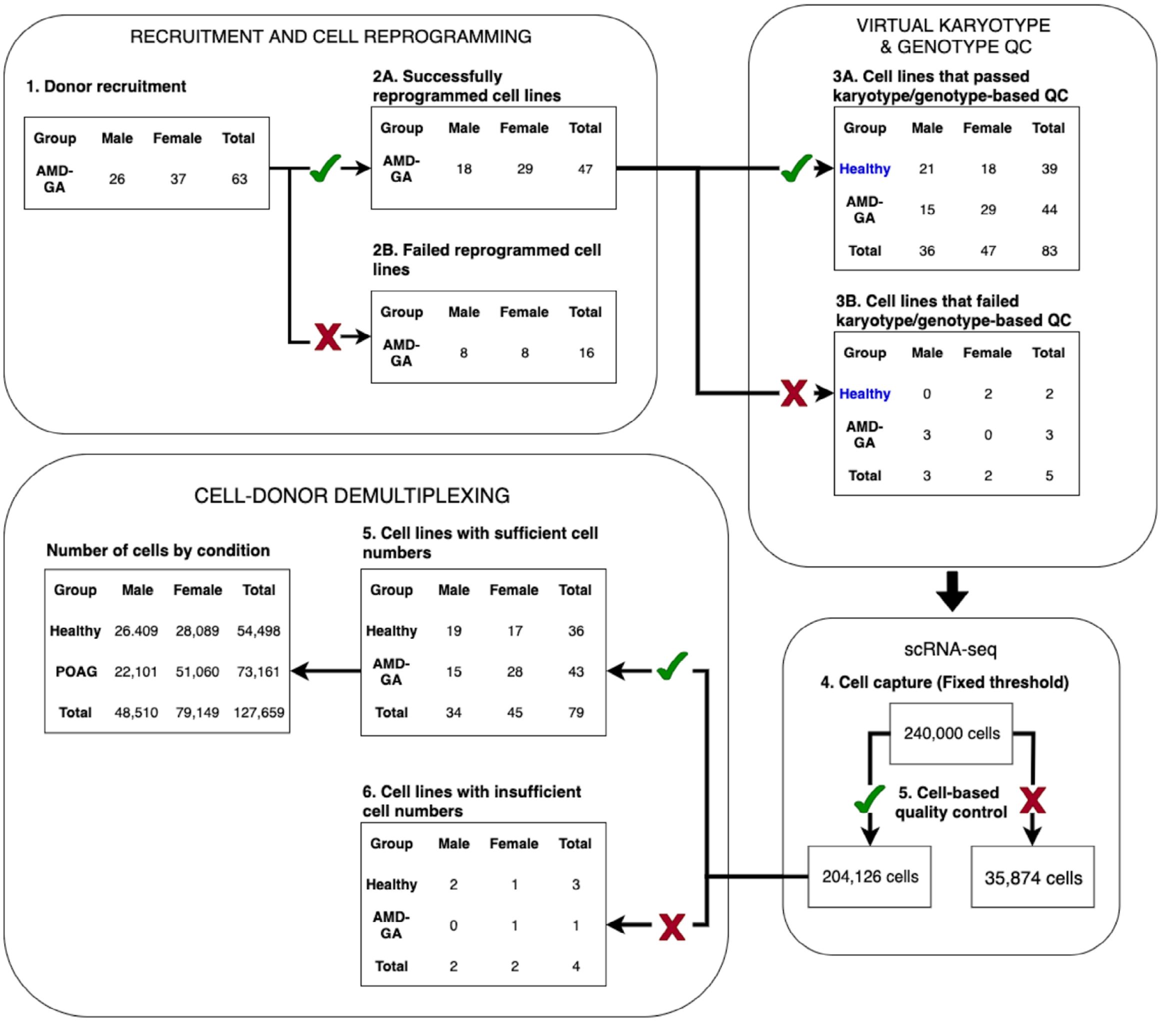

Supplementary Figure 2. Cell line and capture quality control flowchart. Cell lines were generated from skin biopsies of AMD-Geographic atrophy patients, and reprogrammed into iPSCs and subsequently, RPE. Healthy lines were generated in Daniszewski et al (Cell Genomics, 2022). Lines were discarded if they failed virtual karyotype and/or genotype QC, and if less than 200 cells were captured by scRNA-seq.

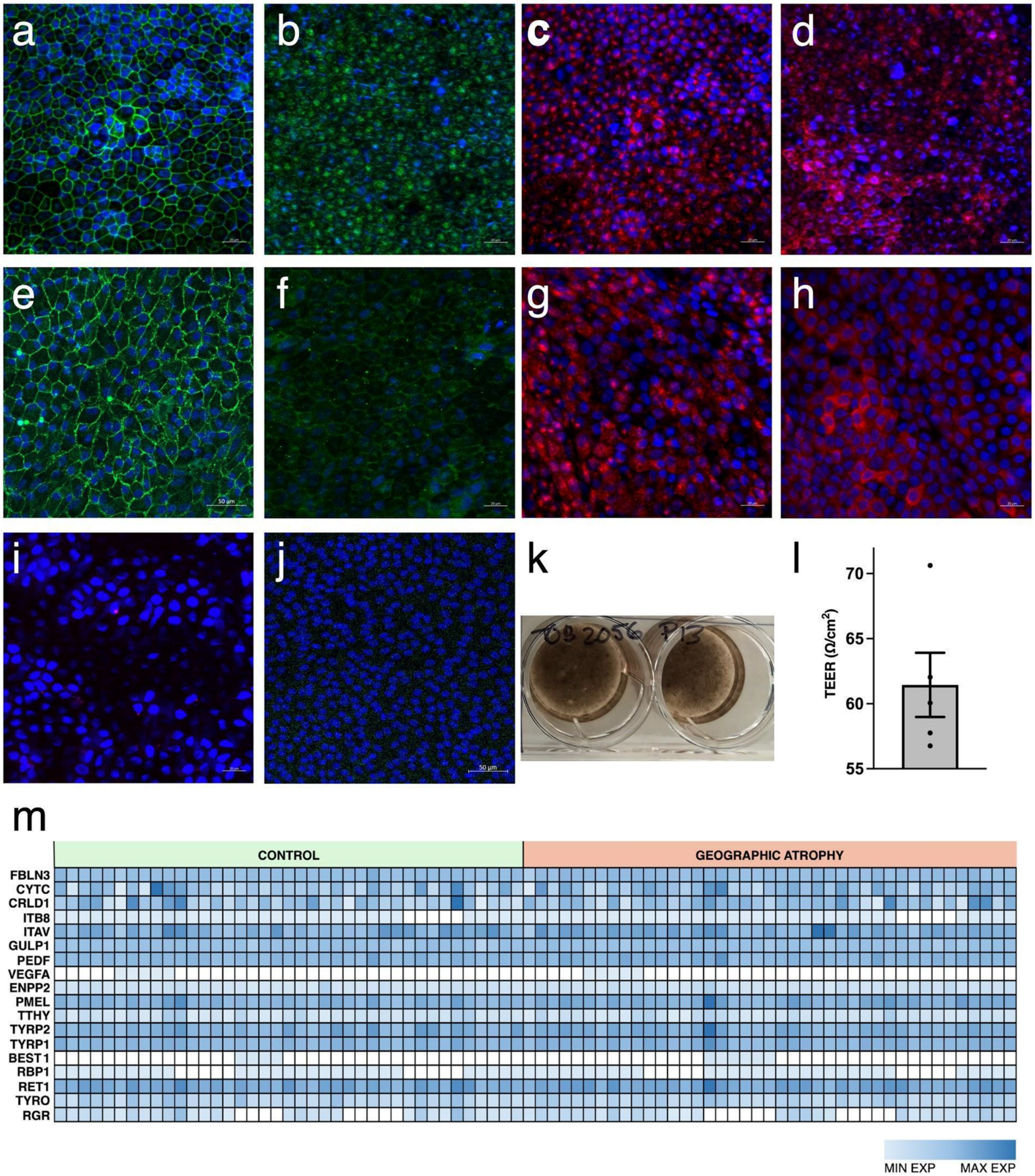

Supplementary Figure 3. Characterisation of RPE cells. (**a-j**) Representative images of control (**a-d**) or geographic atrophy (**e-h**) iPSC-derived RPE cells immunostained with RPE markers ZO-1 (**a, e**), PMEL (**b, f**), BESTROPHIN (**c, g**), RPE65 (**d, h**), or IgG isotype controls for rabbit (**i**) or mouse (**j**) and counterstained with DAPI (Scale bars: **a-d, f-i**: 20 µm; **e:** 50 µm; **j**: 100 µm). (**k**) Representative bright field image of iPSC-derived RPE cells showing cobblestone morphology and pigmentation. (**l**) Transepithelial electrical resistance readings expressed in Ω per cm^2, showing consistency of readings across different cultures. Data are Mean ± SEM of 5 independent wells of a same iPSC-derived RPE cell culture. (**m**) Examples of RPE canonical marker protein expression from the large-scale mass spectrometry proteomic analysis, with all cell lines for each cohort represented as a square. Data presented as a heat map of raw values, with intensity represented by colour gradient (minimal to maximal expression).

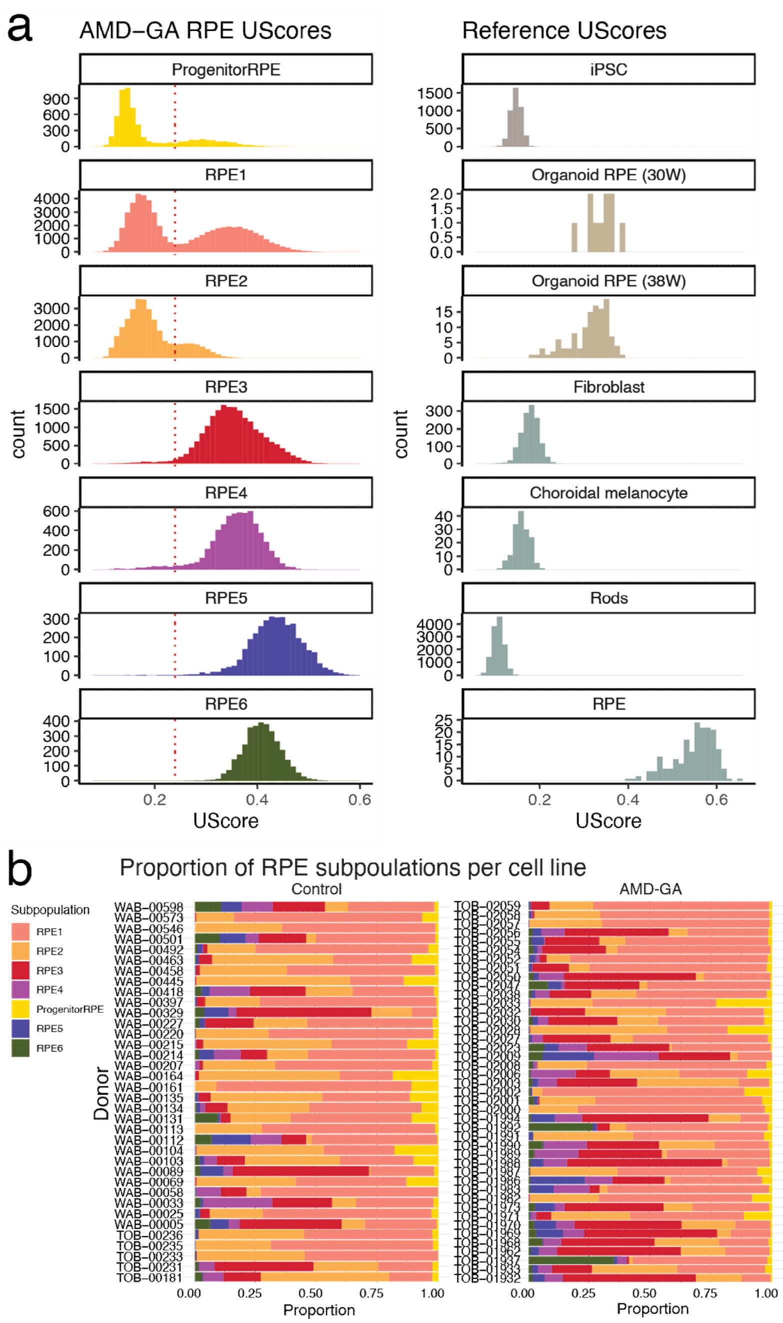

Supplementary Figure 4. Characterisation of the sub populations (a) Distribution of mature RPE gene signature scores in RPE subpopulations and reference datasets. A mature RPE gene signature score - UScore, was calculated for each cell in RPE subpopulation and reference datasets - iPSC (Daniszewski et al. 2018), Organoid - RPE 30W and Organoid - RPE 38W (Cowan et al. 2020), Fibroblasts, Choroidal Melanocytes, Rods and adult RPE (Cowan et al. 2020). Histograms depict distribution of UScore in each tested group. (**b) Proportion of RPE subpopulations per cell line.** Cells were matched to reference RPE subpopulations via cell type classification, and to donors via genotyping. Proportions of RPE subpopulations were calculated for each cell line.

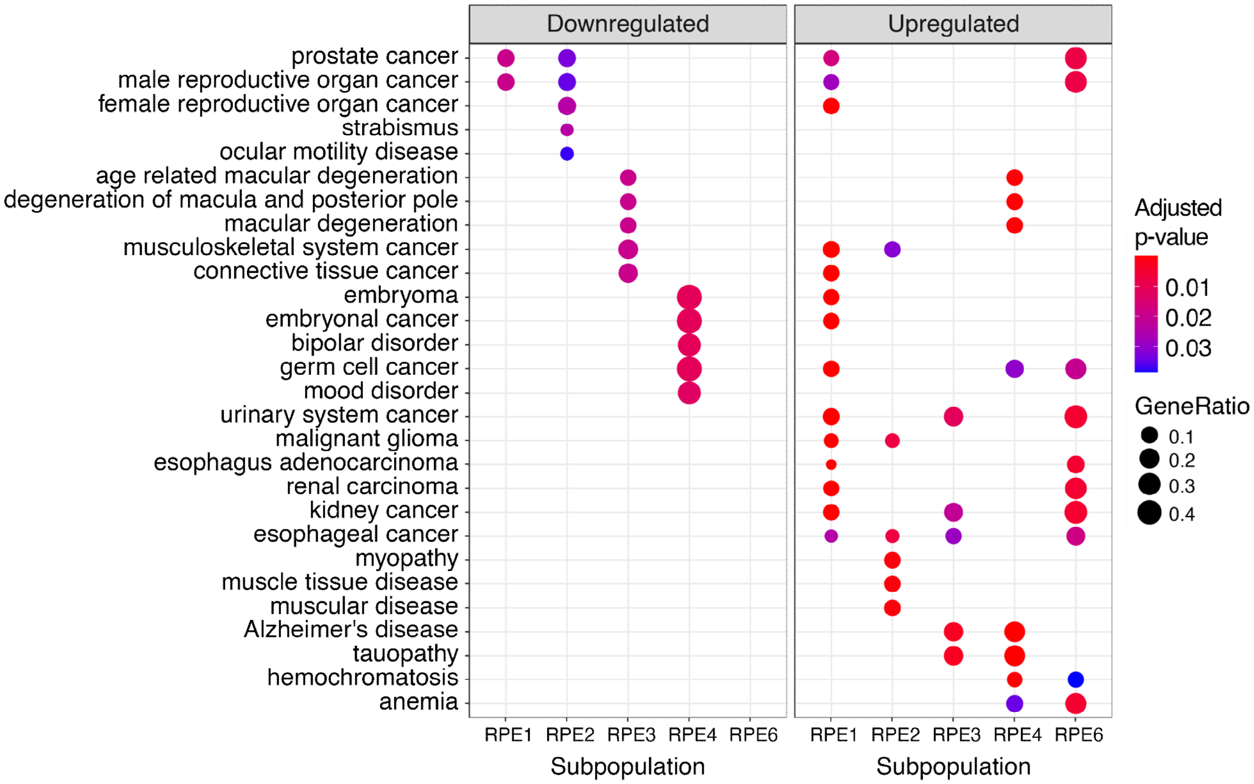

Supplementary Figure 5. Disease ontology analysis. Differentially expressed genes from geographic atrophy cells of each RPE subpopulation underwent over-representation analysis using gene sets from the Disease Ontology database. Upregulated and downregulated genes were analyzed separately. GeneRatio represents the proportion of genes in the query dataset that were also present in the DO gene set. The color scale represents the adjusted p-value of as calculated by the Benjamani & Hochberg method.

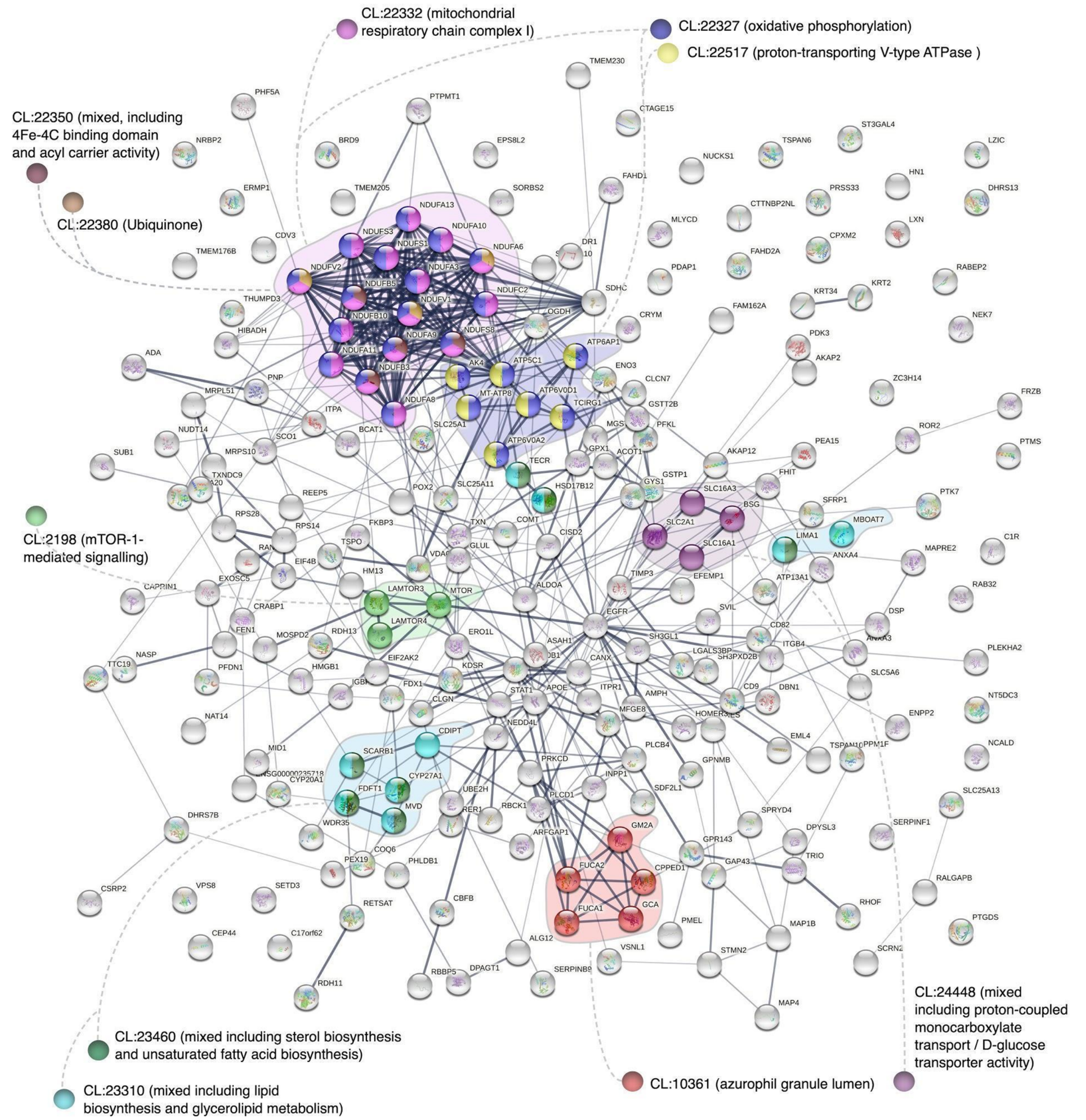

Supplementary Figure 6. Proteome analysis of control and geographic atrophy-RPE cells. Entire local network clustering (STRING) with enriched pathways highlighted in coloured nodules.

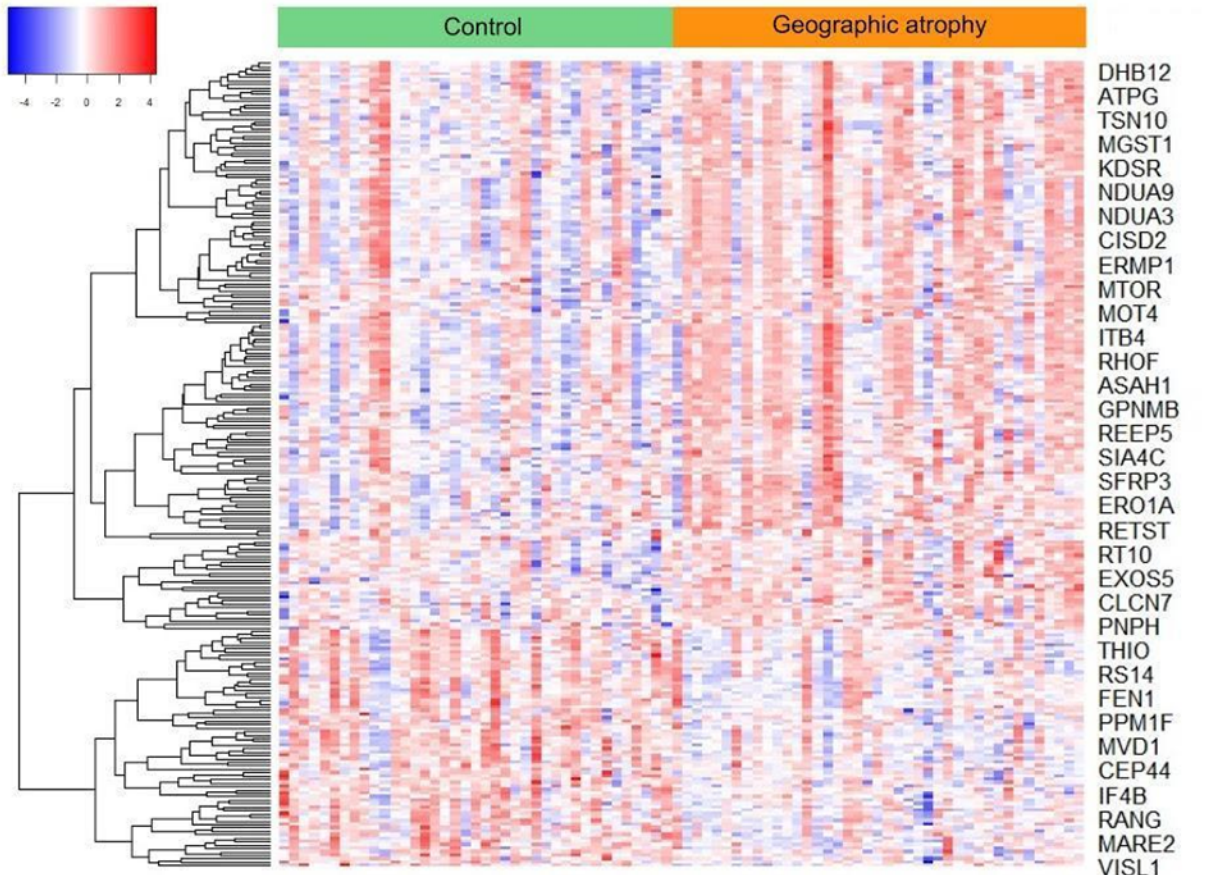

Supplementary Figure 7. Proteome analysis of control and geographic atrophy-RPE cells. Heatmap representation of protein expression in all iPSC-derived RPE cells from each cohort, with expression represented by color gradient.

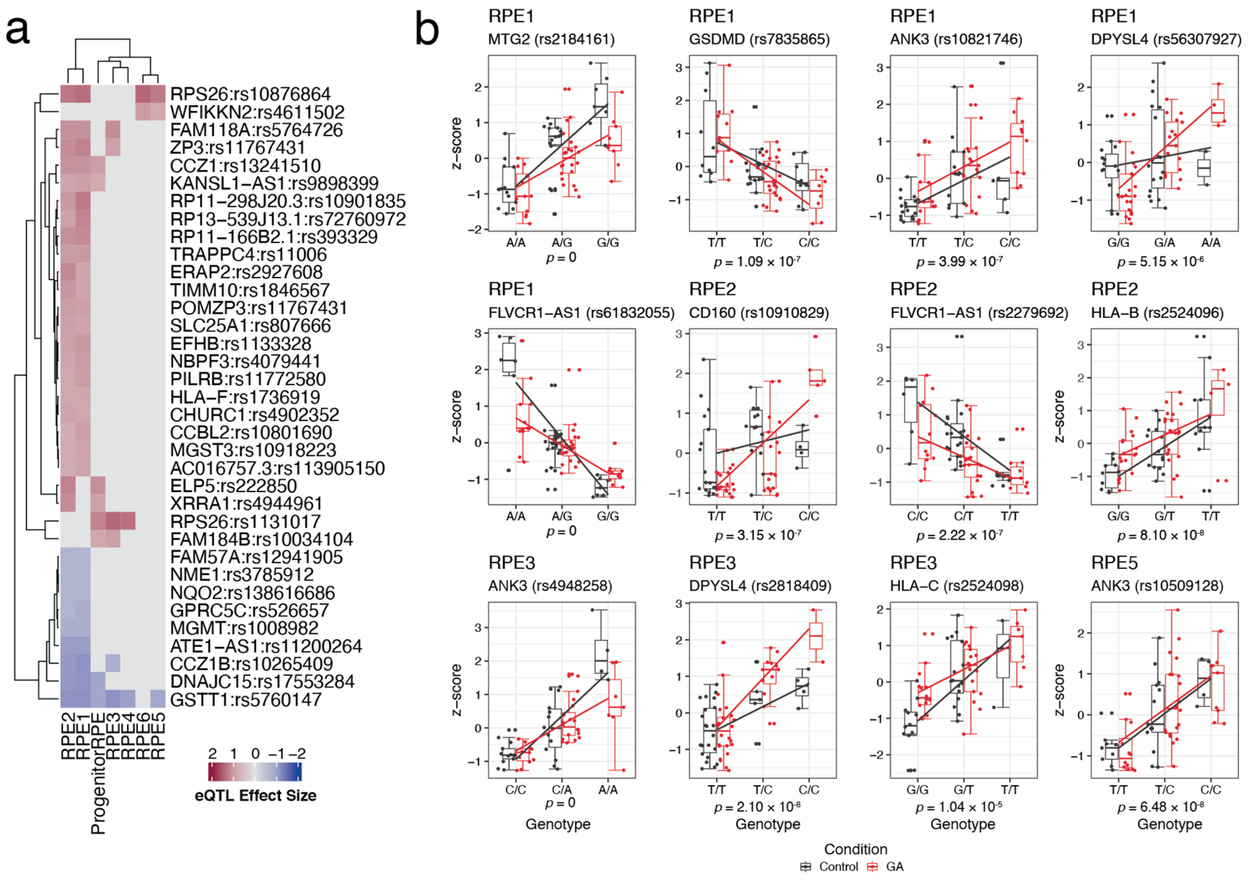

Supplementary Figure 8. **Lead cis-eQTL with known and potential associations with age macular degeneration and geographic atrophy. (a)** Effect sizes and direction of lead cis-eQTL replicated between RPE subpopulations. Effect size is a measure of magnitude of effect of genotype on expression. Red represents effect sizes greater than 0, while blue represents effect sizes below 0. (**b)** To visualize the relationship between genotype and expression, we grouped donors into genotype and condition (Black: Control, Red: Geographic Atrophy) and plotted the aggregated, z-transformed counts of donors as box plots. The slope of the regression line represents changes to the expression trend based on the addition of the alternative allele. All boxplots show p-values for cis-eQTL analysis, medians (horizontal line within box), first (25th percentile) and third (75th percentile) quartiles (lower and upper hinges), 1.5 × the interquartile range (whiskers) and z-scores of individual samples as points. n_{RPE1}: (Control: 35 individuals, GA: 43 individuals), n_{RPE2} = (Control: 35 individuals, GA: 43 individuals), n_{RPE3} = (Control: 31 individuals, GA: 38 individuals), n_{RPE5} = (Control: 26 individuals, GA: 41 individuals).

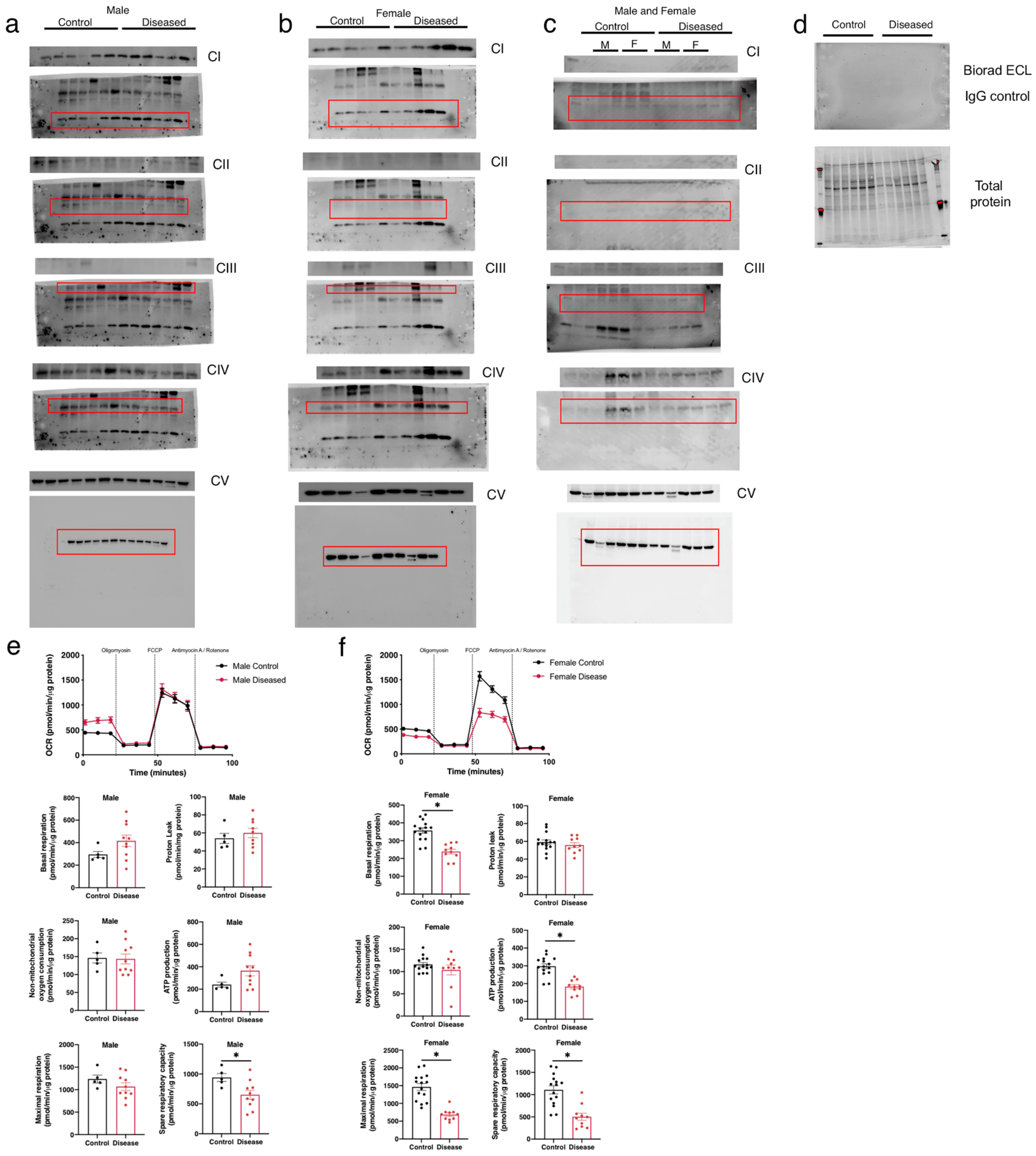

Supplementary Figure 9. Functional assessment of the mitochondria. (**a-c**) Uncropped western blots used for quantifications of Figure 6a, separated into sex (a: male, b: female, corresponding to Figure 6a c: male and female). (**d**) Isotype controls corresponding to the antibodies used for the western blots. (**e-f**) Oxygen consumption rate separated by sex and corresponding to Figure 5b. Quantification of basal respiration, non-mitochondrial oxygen consumption, proton leak, ATP production, maximal respiration and spare respiratory capacity was calculated from 8 biological samples (Control: 1 male, 3 female; geographic atrophy: 2 male, 2 female), containing 5 technical replicates each. Data presented are mean +/- SEM, with statistical significance established as *$p < 0.05$ using two-tailed unpaired *t*-tests. **e:** bottom right panel, male spare respiratory capacity p =

0.0267; **f:** top left panel - female basal respiration p = 0.0000349; bottom left panel - female max respiration p = 0.00000000965; bottom right panel - female spare respiratory capacity p = 0.00000145; middle right panel - female ATP production p = 0.000000667. Source data are provided as a Source Data file.

Supplementary Table 1. Single cell RNA-sequencing quality metrics

Pool	1	2	3	4	5	6	7	8	9	10	11	12
**Forced Number of Cells**	20,000	20,000	20,000	20,000	20,000	20,000	20,000	20,000	20,000	20,000	20,000	20,000
**Mean Reads per Cell**	22,427	22,144	21,305	22,104	22,387	47,134	45,139	45,698	45,068	44,226	45,159	46,388
**Median Genes per Cell**	2,516	2,426	2,519	2556	2,460	3,283	3,049	3,162	3,210	3,052	2,034	3,084
**Number of Reads**	448,558,250	442,888,016	426,111,365	442,084,959	447,748,740	942,691,103	902,785,800	913,970,169	901,377,134	884,523,529	903,190,965	927,769,625
**Valid Barcodes**	97.70%	97.80%	97.70%	97.70%	97.60%	97.50%	97.40%	97.40%	97.50%	97.70%	97.70%	97.40%
**Sequencing Saturation**	29.50%	30.00%	26.60%	25.60%	30.10%	48.60%	45.60%	43.10%	42.40%	50.10%	62.10%	59.10%
**Q30 Bases in Barcode**	96.30%	96.30%	96.30%	96.30%	96.40%	95.80%	95.80%	95.80%	95.80%	95.80%	95.80%	95.80%
**Q30 Bases in RNA Read**	94.80%	94.70%	95.00%	94.90%	95.00%	94.40%	94.10%	94.40%	94.20%	94.00%	94.20%	94.40%
**Q30 Bases in Sample Index**	93.50%	88.40%	94.60%	95.30%	90.30%	94.20%	93.40%	89.80%	89.20%	94.20%	92.10%	94.80%
**Q30 Bases in UMI**	96.20%	96.20%	96.10%	96.10%	96.20%	95.60%	95.50%	95.60%	95.60%	95.60%	95.50%	95.50%
**Reads Mapped to Genome**	96.60%	97.00%	97.00%	96.80%	97.10%	97.30%	96.90%	96.90%	97.00%	97.20%	97.30%	97.60%
**Reads Mapped Confidently to Genome**	93.80%	94.30%	93.50%	93.40%	94.50%	94.80%	94.30%	94.30%	94.60%	95.00%	94.80%	95.20%
**Reads Mapped Confidently to Intergenic Regions**	6.00%	6.20%	5.90%	6.20%	4.90%	5.20%	4.90%	4.70%	4.50%	3.80%	4.10%	4.50%
**Reads Mapped Confidently to Intronic Regions**	28.00%	27.90%	28.60%	30.00%	26.30%	25.30%	25.60%	22.90%	23.60%	22.60%	18.80%	26.20%
**Reads Mapped Confidently to Exonic Regions**	59.80%	60.20%	59.00%	57.20%	63.20%	64.30%	63.70%	66.70%	66.40%	68.60%	71.90%	64.40%
**Reads Mapped Confidently to Transcriptome**	56.20%	56.50%	55.20%	53.40%	59.70%	60.60%	59.80%	62.90%	62.80%	65.60%	68.60%	61.30%
**Reads Mapped Antisense to Gene**	1.50%	1.60%	1.80%	1.70%	1.50%	1.50%	1.80%	1.60%	1.50%	0.90%	1.10%	1.10%
**Fraction Reads in Cells**	86.40%	85.70%	82.50%	83.90%	88.40%	94.10%	90.90%	91.80%	91.90%	91.90%	93.20%	87.70%

Pool	1	2	3	4	5	6	7	8	9	10	11	12
Forced Number of Cells	20,000	20,000	20,000	20,000	20,000	20,000	20,000	20,000	20,000	20,000	20,000	20,000
Mean Reads per Cell	22,427	22,144	21,305	22,104	22,387	47,134	45,139	45,698	45,068	44,226	45,159	46,388
Median Genes per Cell	2,516	2,426	2,519	2556	2,460	3,283	3,049	3,162	3,210	3,052	2,034	3,084
Number of Reads	448,558,250	442,888,016	426,111,365	442,084,959	447,748,740	942,691,103	902,785,800	913,970,169	901,377,134	884,523,529	903,190,965	927,769,625
Valid Barcodes	97.70%	97.80%	97.70%	97.70%	97.60%	97.50%	97.40%	97.40%	97.50%	97.70%	97.70%	97.40%
Sequencing Saturation	29.50%	30.00%	26.60%	25.60%	30.10%	48.60%	45.60%	43.10%	42.40%	50.10%	62.10%	59.10%
Q30 Bases in Barcode	96.30%	96.30%	96.30%	96.30%	96.40%	95.80%	95.80%	95.80%	95.80%	95.80%	95.80%	95.80%
Q30 Bases in RNA Read	94.80%	94.70%	95.00%	94.90%	95.00%	94.40%	94.10%	94.40%	94.20%	94.00%	94.20%	94.40%
Q30 Bases in Sample Index	93.50%	88.40%	94.60%	95.30%	90.30%	94.20%	93.40%	89.80%	89.20%	94.20%	92.10%	94.80%
Q30 Bases in UMI	96.20%	96.20%	96.10%	96.10%	96.20%	95.60%	95.50%	95.60%	95.60%	95.60%	95.50%	95.50%
Reads Mapped to Genome	96.60%	97.00%	97.00%	96.80%	97.10%	97.30%	96.90%	96.90%	97.00%	97.20%	97.30%	97.60%
Reads Mapped Confidently to Genome	93.80%	94.30%	93.50%	93.40%	94.50%	94.80%	94.30%	94.30%	94.60%	95.00%	94.80%	95.20%
Reads Mapped Confidently to Intergenic Regions	6.00%	6.20%	5.90%	6.20%	4.90%	5.20%	4.90%	4.70%	4.50%	3.80%	4.10%	4.50%
Reads Mapped Confidently to Intronic Regions	28.00%	27.90%	28.60%	30.00%	26.30%	25.30%	25.60%	22.90%	23.60%	22.60%	18.80%	26.20%
Reads Mapped Confidently to Exonic Regions	59.80%	60.20%	59.00%	57.20%	63.20%	64.30%	63.70%	66.70%	66.40%	68.60%	71.90%	64.40%
Reads Mapped Confidently to Transcriptome	56.20%	56.50%	55.20%	53.40%	59.70%	60.60%	59.80%	62.90%	62.80%	65.60%	68.60%	61.30%
Reads Mapped Antisense to Gene	1.50%	1.60%	1.80%	1.70%	1.50%	1.50%	1.80%	1.60%	1.50%	0.90%	1.10%	1.10%
Total Genes Detected	25,924	25,469	25,746	26,366	25,710	26,445	26,434	26,274	26,389	24,967	24,826	24,950
Median UMI Counts per Cell	6,970	6,923	6,528	6,507	6,980	11,706	10,698	11,874	11,059	8,699	5,449	8,819

Supplementary Table 2. Deconvolution of shared single cell pools and doublet detection.

Pool	Pooled Individuals	Detected Individuals	Scrublet minimum gene variability percentage	Singlets	Doublets
1	8	8	80	12,891	1,472
2	8	6	80	9,896	2,008
3	8	8	80	11,825	2,398
4	8	8	80	12,559	1,876
5	8	8	80	12,588	1,855
6	8	8	80	11,924	1,699
7	7	7	80	12,419	2,270
8	6	6	80	4,967	2,498
9	7	7	80	12,013	1,862
10	7	8	80	12,843	2,849
11	7	6	80	6,495	1,410
12	6	6	80	9,539	4,602
	88	86	90	129,959	26,799

Supplementary Table 3. Summary of cell type and donor assignments.

Reference	GA	Control	Total	Subpopulation
Common_4	32,254	24,027	56,281	RPE1
Common_6	14,620	16,780	31,400	RPE2
Common_3	14,501	5,851	20,352	RPE3
Common_1	4,329	2,113	6,442	RPE4
Control_3	2,977	3,159	6,136	ProgenitorRPE
Common_2	2,269	1,396	3,665	RPE5
Control_0	2,211	1,172	3,383	RPE6
Control_4	186	98	284	RPE7
Control_2	221	41	262	RPE8
Aged_5	87	36	123	RPE9
Control_5	34	34	68	RPE10
Aged_1	33	21	54	RPE11
Control_1	8	12	20	RPE12
Aged_0	3	0	3	RPE13
Common_5	1	2	3	RPE14
Aged_2	1	0	1	RPE15
Aged_3	0	1	1	RPE16
	73,735	54,743	128,478	

GA: geographic atrophy.

Supplementary Table 4. Post-hoc comparisons of cell subpopulation proportions between conditions

Subpopulation	Residuals (GA)	Adjusted P-values (GA)	Residuals (Control)	Adjusted P-values (Control)
ProgenitorRPE	-1.43E+01	0.00E+00	1.43E+01	0.00E+00
RPE1	-5.43E-03	1.00E+00	5.43E-03	1.00E+00
RPE2	-4.43E+01	0.00E+00	4.43E+01	0.00E+00
RPE3	4.39E+01	0.00E+00	-4.39E+01	0.00E+00
RPE4	1.65E+01	0.00E+00	-1.65E+01	0.00E+00
RPE5	5.71E+00	1.60E-07	-5.71E+00	1.60E-07
RPE6	9.59E+00	0.00E+00	-9.59E+00	0.00E+00

GA: geographic atrophy.

Supplementary Table 5. Statistical significance of differences between average cell subpopulation proportions

Subpopulation	Baseline Prop - Frequency	Prop Mean - GA	Prop Mean - Control	PropRatio	Tstatistic	P.Value	FDR
ProgenitorRPE	0.048	0.036	0.053	0.681	-1.510	0.135	0.207
RPE1	0.441	0.452	0.472	0.957	-0.481	0.632	0.632
RPE2	0.246	0.176	0.284	0.619	-3.095	0.003	0.019
RPE3	0.159	0.215	0.112	1.928	2.427	0.017	0.061
RPE4	0.050	0.057	0.037	1.536	2.024	0.046	0.108
RPE5	0.029	0.035	0.025	1.430	1.462	0.148	0.207
RPE6	0.027	0.029	0.018	1.589	1.234	0.221	0.258

GA: geographic atrophy.

Supplementary Table 6. Replication of single cell-level DE genes in studies of bulk RPE transcriptomes

DE gene set	Bulk DE gene set	Number of common DE genes	% Same effect direction	% Different effect direction
AMD vs Control	Dhirachaikulpanich et al. 2020 - Macula	202	48	52
	Dhirachaikulpanich et al. 2020 - Non-macula	129	53.5	46.5
	Kim et al. 2018 - PRCS	1276	71.7	28.3
Progenitor RPE	Dhirachaikulpanich et al. 2020 - Macula	4	50	50
	Dhirachaikulpanich et al. 2020 - Non-macula	4	25	75.00
	Kim et al. 2018 - PRCS	98	25.5	74.5
RPE1	Dhirachaikulpanich et al. 2020 - Macula	136	54.5	45.6
	Dhirachaikulpanich et al. 2020 - Non-macula	78	50	50
	Kim et al. 2018 - PRCS	887	73.1	26.9
RPE2	Dhirachaikulpanich et al. 2020 - Macula	106	56.6	43.4
	Dhirachaikulpanich et al. 2020 - Non-macula	50	50	50
	Kim et al. 2018 - PRCS	583	75.3	24.7
RPE3	Dhirachaikulpanich et al. 2020 - Macula	23	56.5	43.5
	Dhirachaikulpanich et al. 2020 - Non-macula	7	71.4	28.6
	Kim et al. 2018 - PRCS	105	68.6	31.4
RPE4	Dhirachaikulpanich et al. 2020 - Macula	15	80	20
	Dhirachaikulpanich et al. 2020 - Non-macula	1	100	0
	Kim et al. 2018 - PRCS	70	85.7	14.3

RPE5	Dhirachaikulpanich et al. 2020 - Macula	10	70	30
	Dhirachaikulpanich et al. 2020 - Non-macula	4	75	25
	Kim et al. 2018 - PRCS	36	27.8	72.2
RPE6	Dhirachaikulpanich et al. 2020 - Macula	6	16.7	83.3
	Dhirachaikulpanich et al. 2020 - Non-macula	2	0	100
	Kim et al. 2018 - PRCS	44	47.7	52.3

Supplementary Table 7. Percentage replication of lead cis-eQTL between RPE subpopulations.

**Subpopulation**	**ProgenitorRPE**	**RPE1**	**RPE2**	**RPE3**	**RPE4**	**RPE5**	**RPE6**
**ProgenitorRPE**	100	1.7	5.1	7.9	50	4.3	0
**RPE1**	11.1	100	25.4	10.5	25	8.7	16.7
**RPE2**	16.7	13	100	10.5	25	8.7	16.7
**RPE3**	8.3	1.7	3.4	100	50	4.3	0
**RPE4**	5.6	0.4	0.8	5.3	100	4.3	0
**RPE5**	2.8	0.9	1.7	2.6	25	100	33.3
**RPE6**	0	0.4	0.8	0	0	8.7	100

Supplementary Table 8. Common cis-eQTL in RPE1 and RPE2

Gene	rsID	EffectSize eQTL (RPE1)	EffectSize Interaction (RPE1)	EffectSize eQTL (RPE2)	EffectSize Interaction (RPE2)
RPS26	rs10876864	1.38	0.31	1.30	0.16
RP11-298J20.3	rs10901835	1.28	-0.03	1.03	-0.06
CCZ1B	rs10265409	-1.18	0.04	-1.11	-0.30
ZP3	rs11767431	1.19	0.33	1.11	0.38
RP13-539J13.1	rs72760972	1.17	0.05	0.93	0.05
GSTT1	rs5760147	-1.18	-0.37	-1.17	-0.20
DNAJC15	rs17553284	-1.16	0.26	-1.11	0.15
RP11-166B2.1	rs393329	1.13	-0.04	1.09	-0.38
CCZ1	rs13241510	1.06	0.15	1.07	0.10
TRAPPC4	rs11006	1.02	0.11	1.01	0.20
FAM118A	rs5764726	1.09	0.50	1.10	0.33
KANSL1-AS1	rs9898399	0.96	-0.30	0.87	-0.42
PILRB	rs11772580	0.98	0.42	0.82	-0.22
CCBL2	rs10801690	0.94	-0.45	0.75	-0.13
ATE1-AS1	rs11200264	-1.06	0.23	-1.05	0.07
EFHB	rs1133328	1.01	0.43	0.93	0.46
POMZP3	rs11767431	0.94	-0.04	0.98	-0.30
GPRC5C	rs526657	-0.87	0.10	-0.77	-0.36
SLC25A1	rs807666	0.89	0.40	0.94	0.35
NBPF3	rs4079441	1.01	0.39	0.87	0.32
MGST3	rs10918223	0.88	-0.08	0.78	0.01
TIMM10	rs1846567	0.87	-0.17	1.04	-0.45
MGMT	rs1008982	-0.86	0.04	-0.87	-0.23
AC016757.3	rs113905150	0.86	-0.27	0.77	-0.24
ERAP2	rs2927608	0.86	0.19	1.14	0.29
NME1	rs3785912	-0.77	-0.43	-0.74	-0.25
CHURC1	rs4902352	0.82	-0.06	0.87	0.49
FAM57A	rs12941905	-0.74	0.30	-0.76	0.02
NQO2	rs138616686	-0.78	0.35	-0.74	0.11
HLA-F	rs1736919	0.86	0.49	0.83	0.24
