# Supplementary material for: Transcriptomic and proteomic retinal pigment epithelium signatures of age-related macular degeneration
Source: Nat Commun. 2022 Jul 26;13:4233. doi: 10.1038/s41467-022-31707-4 (PMC9325891; doi:10.1038/s41467-022-31707-4)
Supplement: Supplementary file 2 — Description of Additional Supplementary Files [file 41467_2022_31707_MOESM2_ESM.pdf]

Description of Additional Supplementary Files

File Name: Supplementary Data 1
Description: Registry of individual lines; disease loci genotypes; RPE gene sets; markers of each subpopulation and proteomics analysis dataset.

File Name: Supplementary Data 2
Description: Analyses of differentially expressed (DE) genes.

File Name: Supplementary Data 3
Description: Analyses of cis-eQTL, cis-pQTL and transcriptome-wide association study.
