# Supplementary material for: Transcriptomic and proteomic retinal pigment epithelium signatures of age-related macular degeneration
Source: Nat Commun. 2022 Jul 26;13:4233. doi: 10.1038/s41467-022-31707-4 (PMC9325891; doi:10.1038/s41467-022-31707-4)
Supplement: Supplementary file 6 — Reporting Summary [file 41467_2022_31707_MOESM6_ESM.pdf]

nature portfolio

Corresponding author(s): Alice Pébay

Last updated by author(s): 31/05/22

Reporting Summary

Nature Portfolio wishes to improve the reproducibility of the work that we publish. This form provides structure for consistency and transparency in reporting. For further information on Nature Portfolio policies, see our Editorial Policies and the Editorial Policy Checklist.

Statistics

For all statistical analyses, confirm that the following items are present in the figure legend, table legend, main text, or Methods section.

n/a	Confirmed	
☐	☒	The exact sample size (*n*) for each experimental group/condition, given as a discrete number and unit of measurement
☐	☒	A statement on whether measurements were taken from distinct samples or whether the same sample was measured repeatedly
☐	☒	The statistical test(s) used AND whether they are one- or two-sided<br>*Only common tests should be described solely by name; describe more complex techniques in the Methods section.*
☐	☒	A description of all covariates tested
☐	☒	A description of any assumptions or corrections, such as tests of normality and adjustment for multiple comparisons
☐	☒	A full description of the statistical parameters including central tendency (e.g. means) or other basic estimates (e.g. regression coefficient) AND variation (e.g. standard deviation) or associated estimates of uncertainty (e.g. confidence intervals)
☐	☒	For null hypothesis testing, the test statistic (e.g. *F*, *t*, *r*) with confidence intervals, effect sizes, degrees of freedom and *P* value noted<br>*Give P values as exact values whenever suitable.*
☒	☐	For Bayesian analysis, information on the choice of priors and Markov chain Monte Carlo settings
☐	☒	For hierarchical and complex designs, identification of the appropriate level for tests and full reporting of outcomes
☐	☒	Estimates of effect sizes (e.g. Cohen's *d*, Pearson's *r*), indicating how they were calculated

Our web collection on statistics for biologists contains articles on many of the points above.

Software and code

Policy information about availability of computer code

Data collection	Single cell RNA-sequencing data was generated using the Cell Ranger Software Suite v3.10 by 10x Genomics. Genotype data was processed using GenomeStudio PLINK Input Report Plug-in v 2.14. Proteomics data was generated with MSConvert v3. Images were acquired on a Zeiss Axio Imager M2 fluorescent microscope using ZEN Blue 3.5 software.
Data analysis	Single cell RNA-sequencing data was analysed using popscle v0.1-beta, souporcell v1.2.0, scrublet v.0.2.1, DoubletDetection v2.5.2 and R v3 and v4.05. R was used with the following R packages: Seurat (v3.1.5, v4.05), scPred v0.9, clusterProfiler v1.9, and MAST v1.16. eQTL analysis was performed with MatrixEQTL v2.3 and pQTL analysis was performed with QTLtools v1.3.1. TWAS analysis was performed with FUSION TWAS 2019/10/01 (https://github.com/gusevlab/fusion_twas). Proteomics used the STRING database version 11.0. STRING analysis Cellular and mitochondrial analyses used ImageLab software Version 4.1, Fiji/ImageJ software Version 2.0, Graphpad prism v9.

For manuscripts utilizing custom algorithms or software that are central to the research but not yet described in published literature, software must be made available to editors and reviewers. We strongly encourage code deposition in a community repository (e.g. GitHub). See the Nature Portfolio guidelines for submitting code & software for further information.

Data

Policy information about availability of data

All manuscripts must include a data availability statement. This statement should provide the following information, where applicable:

- Accession codes, unique identifiers, or web links for publicly available datasets
- A description of any restrictions on data availability
- For clinical datasets or third party data, please ensure that the statement adheres to our policy

The transcriptomic data were mapped to the Homo sapiens reference hg19/GRCh37 from ENSEMBL (release 75) (https://grch37.ensembl.org/index.html). SNP

genotype imputation used the Haplotype Reference Consortium panel (HRC r1.1 2016) (http://www.haplotype-reference-consortium.org/).The proteomics analysis used the STRING database version 11.0. STRING analysis (https://string-db.org) and the Human UniProt Database (https://www.uniprot.org). We used the Gene Ontology (ref67,134) and Disease Ontology (ref 135) databases.

The transcriptomic data generated in this study have been deposited in the ArrayExpress database under accession code E-MTAB-11642 (https://www.ebi.ac.uk/arrayexpress). The mass spectrometry raw file and search results have been deposited to the ProteomeXchange Consortium via the PRIDE 144 partner repository with the dataset identifier PXD029501 (http://www.ebi.ac.uk/pride).

Field-specific reporting

Please select the one below that is the best fit for your research. If you are not sure, read the appropriate sections before making your selection.

☒ Life sciences ☐ Behavioural & social sciences ☐ Ecological, evolutionary & environmental sciences

For a reference copy of the document with all sections, see nature.com/documents/nr-reporting-summary-flat.pdf

Life sciences study design

All studies must disclose on these points even when the disclosure is negative.

Sample size

We reprogrammed fibroblasts into iPSCs from 63 individuals with geographic atrophy (all of Northern European descent, 37 female and 26 male; mean ± SD age at recruitment: 83.8 ± 8.2 years) using episomal vectors, with lines from 47 individuals successfully reprogrammed . We matched these iPSCs with control iPSC lines from ethnically- matched healthy individuals that were generated and characterized in a previous study . Each line was genotyped for 787,443 single nucleotide polymorphisms (SNPs) and imputed with the Haplotype Reference Consortium panel After quality control, this yielded 4,309,001 autosomal SNPs with MAF >10%.
This is the largest iPSC study for the modelling of AMD/ geographic atrophy.

Data exclusions

To ensure a diagnosis of AMD and not a monogenic retinal disease causing atrophy, all case participants had drusen identified on clinical examination. Dominantly inherited drusen phenotypes such as Sorsby fundus dystrophy, Doyne's honeycomb dystrophy and Malattia Leventinese as well as fleck dystrophies such as Stargardt's disease were excluded.
The differentiation of all iPSC lines to RPE was performed in two large independent differentiation batches, and lines that did not differentiate sufficiently to RPE were discarded from analysis, which was based on clear cobblestone morphology of the cells and pigmentation throughout the culture.

Replication

For sequencing and mass spectrometry/ proteomic analysis, each cohort (control and disease) had n=43 and n=36 independent lines respectively.
Westernblot analysis was performed on 17 cell lines on two independent experiments.
For seahorse analysis, the Seahorse Analyzer was run using 9-min cyclic protocol commands (mix for 3 min, stand for 3 min, and measure for 3 min) in triplicate. Real-time oxygen consumption rate assay was performed on 4 cell lines on two independent experiments, with five technical replicates.

Randomization

Samples from the control and disease cohorts were run side by side for each differentiation batch. The differentiation of all iPSC lines to RPE was performed in two large independent differentiation batches that were independent of disease status and lines that did not differentiate sufficiently to RPE were discarded from analysis. For RNA Sequencing, differentiated cell lines were randomly divided into 12 pools that each consisted of up to 8 cell lines from both control and AMD groups. For mass spectrometry, all samples were were run through 8 independent TMT experiments, with equal number of samples from both cohorts for each TMT.

Blinding

All lines were coded and investigators were blind to their identity during the experimental phase. The uncoding/ donor assignment only took place at analysis when groups needed to be defined (ie once single cell transcriptome and proteomic profiles were generated. For proteomic analysis, the two cohorts were colored coded (1 group green 1 group white) and the uncoding of conditions was only performed post analysis. For the western blot and sehorse analysis, the investigator performing the experiment was blinded to the identity of the samples, those were decoded post experiments once data were generated.

Reporting for specific materials, systems and methods

We require information from authors about some types of materials, experimental systems and methods used in many studies. Here, indicate whether each material, system or method listed is relevant to your study. If you are not sure if a list item applies to your research, read the appropriate section before selecting a response.

Materials & experimental systems

n/a	Involved in the study
☐	☒ Antibodies
☐	☒ Eukaryotic cell lines
☒	☐ Palaeontology and archaeology
☒	☐ Animals and other organisms
☐	☒ Human research participants
☒	☐ Clinical data
☒	☐ Dual use research of concern

Methods

n/a	Involved in the study
☒	☐ ChIP-seq
☒	☐ Flow cytometry
☒	☐ MRI-based neuroimaging

Antibodies

Antibodies used

OCT3/4 (sc-5279, dil 1/40, Santa Cruz Biotechnology), TRA-1-60 (MA1-023-PE, dil 1/100, Thermo Fisher Scientific), ZO-1 (#339100, 10µg/mL, Life Technologies), PMEL (ab137062, 5µg/mL, Abcam), BESTROPHIN (ab2182, 2µg/mL, Abcam), OCCLUDIN (33-1500 (OC-3F10), 3µg/mL, Thermo Fisher), RPE 65 (ab235950, 10µg/mL, Abcam); Total OXPHOS (MS604-300, 10µg/mL, Abcam 110413); Alexa Fluor 568 Goat Anti-Mouse IgG, # A-11031, 1:1000; and Alexa Fluor 488 Goat Anti-Mouse IgG, # A-11029, 1:1000 both from Thermo Fisher Scientific), HRP-conjugated sheep anti-mouse IgG antibody (NA9310V, GE Healthcare)

Validation

All antibodies have been widely used and validated.
Validation of all antibodies were taken from the manufacturers' websites

OCT3/4: https://datasheets.scbt.com/sc-5279.pdf
"Oct-3/4 (C-10) is recommended for detection of Oct-3/4 of mouse, rat and human origin by Western Blotting (starting dilution 1:200, dilution range 1:100-1:1000), immunoprecipitation [1-2 µg per 100-500 µg of total protein (1 ml of cell lysate)], immunofluorescence (starting dilution 1:50, dilution range 1:50-1:500), immunohistochemistry (including paraffin-embedded sections) (starting dilution 1:50, dilution range 1:50-1:500), flow cytometry (1 µg per 1 x 10 6 cells) and solid phase ELISA (starting dilution 1:30, dilution range 1:30-1:3000); non cross-reactive with Oct-3/4 isoform B."

TRA160: https://www.thermofisher.com/antibody/product/TRA-1-60-Antibody-clone-TRA-1-60-Monoclonal/MA1-023-PE;
"TRA-1-60 is a cell surface antigen, expressed along with SSEA-3, SSEA-4 and TRA-1-81 in human embryonic stem cells, embryonal carcinoma cells and induced pluripotent stem cells (iPS). These surface markers are lost during the differentiation process. In contrast, SSEA-1 is absent in undifferentiated human stem cells but is present on the cell surface after retinoic acid mediated differentiation."

ZO-1: https://www.thermofisher.com/order/genome-database/details/antibody/33-9100.html "Advanced Verification This Antibody was verified by Knockdown to ensure that the antibody binds to the antigen stated".

PMEL: https://www.abcam.com/melanoma-gp100-antibody-epr4864-ab137062.html
"Suitable for: WB, IHC-P Species reactivity Reacts with: Human
...
This product is a recombinant monoclonal antibody, which offers several advantages including:- High batch-to-batch consistency and reproducibility; - Improved sensitivity and specificity"

BESTROPHIN https://www.abcam.com/bestrophinbest1-antibody-e6-6-ab2182.html
"Key features and details: Mouse monoclonal [E6-6] to Bestrophin/BEST1, Suitable for: WB, IHC-Fr, ICC/IF, IP, Reacts with: Cow, Dog, Human, Pig, Monkey"

OCCLUDIN https://www.thermofisher.com/antibody/product/Occludin-Antibody-clone-OC-3F10-Monoclonal/33-1500
"Advanced Verification This Antibody was verified by Knockdown to ensure that the antibody binds to the antigen stated".

RPE 65: https://www.abcam.com/rpe65-antibody-ab235950.html
Recombinant fragment corresponding to Human RPE65 aa 300-500.
Rabbit polyclonal to RPE65, Suitable for: WB, ICC/IF, Reacts with: Rat, Human

Total OXPHOS (MS604-300, 10µg/mL, Abcam ab110413) https://www.abcam.com/total-oxphos-rodent-wb-antibody-cocktail-ab110413.html
Reacts with: Mouse, Rat, Cow, Human, Cynomolgus monkey
Total OXPHOS Rodent WB Antibody Cocktail ab110413 is an optimized cocktail of high quality antibodies for analyzing relative levels of OXPHOS complexes in rat or mouse mitochondria by western blot. This OXPHOS cocktail contains 5 mouse mAbs, one each against CI subunit NDUFB8 (ab110242), CII-30kDa (ab14714), CIII-Core protein 2 (ab14745), CIV subunit I (ab14705) and CV alpha subunit (ab14748) as an optimized premixed cocktail. The kit is suitable for Western Blotting analysis of the relative levels of the 5 OXPHOS complexes in mitochondrial preparations from mouse, rat, human, or bovine sources.

Eukaryotic cell lines

Policy information about cell lines

Cell line source(s)	All Geographic atrophy iPSC lines were generated in this study. All control lines were generated and described in our previous study (Daniszewski et al Cell Genomics 2022.
Authentication	None of the cell lines used were authenticated.
Mycoplasma contamination	All lines were tested for mycoplasma and were negative.
Commonly misidentified lines (See ICLAC register)	*Name any commonly misidentified cell lines used in the study and provide a rationale for their use.*

Human research participants

Policy information about studies involving human research participants

Population characteristics	As per Supp Fig 2, we reprogrammed fibroblasts into iPSCs from 63 individuals with advanced AMD with geographic atrophy in at least one eye and an age at first diagnosis over 50 years (all of Northern European descent, 37 female and 26 male; mean ± SD age at recruitment: 83.8 ± 8.2 years) of which 44 were successfully reprogrammed to iPSCs (15 male, 29 female). We matched these iPSCs with 39 control iPSC lines from ethnically- matched healthy individuals that were generated and characterized in a previous study (Daniszewski et al Cell Genomics 2022, mean ± SD age at recruitment for participants was 69.8 ± 9.5 years, 21 males, 18 females) and had no manifest ophthalmic disease or drusen. Post quality control, we assessed 43 geographic atrophy lines (73,161 cells, 15 males, 28 females, 83.4 ± 8.6 years) and 36 control lines (54,498 cells, 19 males, 17 females, mean ± SD age of samples 67.6 ± 9.5 years).
Recruitment	Cases who had advanced AMD with geographic atrophy in at least one eye and an age at first diagnosis over 50 years, were recruited through local ophthalmic clinics. Participants in the control cohort had no manifest ophthalmic disease or drusen. To ensure a diagnosis of AMD and not a monogenic retinal disease causing atrophy, all case participants had drusen identified on clinical examination. Dominantly inherited drusen phenotypes such as Sorsby fundus dystrophy, Doyne's honeycomb dystrophy and Malattia Leventinese as well as fleck dystrophies such as Stargardt's disease were excluded. Our cohort is biased by the fact that all participants were genetically defined as being of Northern European descent, and as such the results may not reflect people from other populations.
Ethics oversight	All participants gave informed written consent. This study was approved by the Human Research Ethics committees of the Royal Victorian Eye and Ear Hospital (11/1031H, 13/1151H-004), University of Melbourne (1545394), University of Tasmania (H0014124) UWA (EPS) as per the requirements of the NHMRC, in accordance with the Declarations of Helsinki.

Note that full information on the approval of the study protocol must also be provided in the manuscript.
